# *Aspergillus fumigatus* secondary metabolite pyripyropene is important for the dual biofilm formation with *Pseudomonas aeruginosa*

**DOI:** 10.1128/mbio.00363-25

**Published:** 2025-03-17

**Authors:** Patricia Alves de Castro, Daniel Yuri Akiyama, Camila Figueiredo Pinzan, Thaila Fernanda dos Reis, Endrews Delbaje, Peter Rocha, Mario Augusto Izidoro, Sérgio Schenkman, Shinya Sugimoto, Norio Takeshita, Karin Steffen, Jessica L. Aycock, Stephen K. Dolan, Antonis Rokas, Taícia Fill, Gustavo H. Goldman

**Affiliations:** 1 Faculdade de Ciências Farmacêuticas de Ribeirão Preto, Universidade de São Paulo, Ribeirão Preto, Brazil; 2 Instituto de Química, Universidade Estadual de Campinas 28132 , Campinas, Brazil; 3 Hospital São Paulo, Vila Clementino, São Paulo, Brazil; 4 Departamento de Microbiologia, Imunologia e Parasitologia, Escola Paulista de Medicina, Universidade Federal de São Paulo, São Paulo, Brazil; 5 Department of Bacteriology, Jikei Center for Biofilm Science and Technology, Laboratory of Amyloid Regulation, The Jikei University School of Medicine, Tokyo, Japan; 6 Microbiology Research Center for Sustainability (MiCS), Faculty of Life and Environmental Sciences, University of Tsukuba, Tsukuba, Japan; 7 Department of Biological Sciences, Vanderbilt University, Nashville, Tennessee, USA; 8 Evolutionary Studies Initiative, Vanderbilt University, Nashville, Tennessee, USA; 9 Department of Genetics and Biochemistry, Eukaryotic Pathogens Innovation Center, Clemson University, Clemson, South Carolina, USA; 10 National Institute of Science and Technology in Human Pathogenic Fungi, São Paulo, Brazil; Institut Pasteur, Paris, France

**Keywords:** *Aspergillus fumigatus*, *Pseudomonas aeruginosa*, biofilm formation, G-protein signaling, secondary metabolites, pyripyropene

## Abstract

The human pathogenic fungus *Aspergillus fumigatus* establishes dual biofilm interactions in the lungs with the pathogenic bacterium *Pseudomonas aeruginosa*. Screening of 21 *A*. *fumigatus* null mutants revealed seven mutants (two G protein-coupled receptors, three mitogen-activated protein kinase receptors, a Gα protein, and one histidine kinase receptor) with reduced biofilm formation, specifically in the presence of *P. aeruginosa*. Transcriptional profiling and metabolomics analysis of secondary metabolites produced by one of these mutants, Δ*gpaB* (*gpaB* encodes a Gα protein), showed GpaB controls the production of several important metabolites for the dual biofilm interaction, including pyripyropene A, a potent inhibitor of mammalian acyl-CoA cholesterol acyltransferase. Deletion of *pyr2*, encoding a non-reducing polyketide synthase essential for pyripyropene biosynthesis, showed reduced *A. fumigatus* Δ*pyr2-P. aeruginosa* biofilm growth, altered macrophage responses, and attenuated mouse virulence in a chemotherapeutic murine model. We identified pyripyropene as a novel player in the ecology and pathogenic interactions of this important human fungal pathogen.

**IMPORTANCE:**

*Aspergillus fumigatus* and *Pseudomonas aeruginosa* are two important human pathogens. Both organisms establish biofilm interactions in patients affected with chronic lung pulmonary infections, such as cystic fibrosis (CF) and chronic obstructive pulmonary disease. Colonization with *A. fumigatus* is associated with an increased risk of *P. aeruginosa* colonization in CF patients, and disease prognosis is poor when both pathogens are present. Here, we identified *A. fumigatus* genetic determinants important for the establishment of *in vitro* dual *A. fumigatus-P. aeruginosa* biofilm interactions. Among them, an *A. fumigatus* Gα protein GpaB is important for this interaction controlling the production of the secondary metabolite pyripyropene. We demonstrate that the lack of pyripyropene production decreases the dual biofilm interaction between the two species as well as the virulence of *A. fumigatus* in a chemotherapeutic murine model of aspergillosis. These results reveal a complete novel role for this secondary metabolite in the ecology and pathogenic interactions of this important human fungal pathogen.

## INTRODUCTION

*Aspergillus fumigatus* is a filamentous fungus found in soil, water, air, decaying organic matter, and plant-based products (1, 2). Aspergillosis is a group of diseases caused by *A. fumigatus* that range from chronic or hypersensitization (allergic responses) disorders to invasive and life-threatening infections (3). Cystic fibrosis (CF) is a genetic illness characterized by mutations in the CF transmembrane conductance regulator gene, which results in faulty chloride secretion, altered airway surface fluids, ciliary dyskinesis, and reduced mucociliary clearance (4). Such modifications result in immobile mucus plaques, which produce a hypoxic environment conducive to the colonization and survival of numerous fungi and bacteria, including *A. fumigatus* and *Pseudomonas aeruginosa* (5, 6). Notably, the presence of *A. fumigatus* in respiratory CF samples has been linked to a worse prognosis and a deterioration in pulmonary function (7). *P. aeruginosa* is a versatile gram-negative bacterium capable of thriving in both aerobic and anaerobic conditions. This species is prevalent throughout nature, inhabiting soil and water as well as invading humans, where it can behave as an opportunistic pathogen (6).

There is clinical evidence that *A. fumigatus* and *P. aeruginosa* cocolonize CF patients, which is linked to a reduction in lung function. It has been reported that 60% of individuals with chronic *P. aeruginosa* infection also have *A. fumigatus* (8–11). Although several of these studies have looked at this interaction *in vitro,* few have looked at how *A. fumigatus* and *P. aeruginosa* interact *in vivo* and the consequences for the host (6, 12–17). There is also a paucity of research identifying fungal and bacterial genes expressed and the secondary metabolites (SMs) present during coculturing and mixed biofilms, as well as their involvement in the fungus-bacterium interaction. We recently annotated fungal SMs and bacterial compounds produced in mixed *A. fumigatus-P. aeruginosa* biofilms grown in normoxia and hypoxia (18). *P. aeruginosa* produced nine compounds, which we identified as phenazines and various analogs of pyoverdine, and the fungal presence boosted their secretion levels. The roles of the two phenazine-producing operons (*phzA1* and *phzA2*) were also studied, and mutants lacking one of those operons were able to create incomplete sets of phenazines (18). We additionally found 20 SMs secreted by *A. fumigatus* in either monoculture or coculture with *P. aeruginosa*. All these compounds were secreted during biofilm formation in either normoxia or hypoxia. However, only eight compounds (demethoxyfumitremorgin C, fumitremorgin, ferrichrome, ferricrocin, triacetylfusigen, gliotoxin, gliotoxin E, and pyripyropene A) were detected during biofilm formation by the coculture of *A. fumigatus* and *P. aeruginosa* under normoxia and hypoxia conditions (18). Overall, we showed how diverse SM secretion is during *A. fumigatus* and *P. aeruginosa* mixed culture and how this can affect biofilm formation in normoxia and hypoxia (18).

Here, we extended these studies by screening null mutants of genes encoding proteins involved in surface recognition, such as G protein-coupled receptors (GPCRs), mitogen-activated protein kinase (MAPK) receptors, a histidine kinase receptor, and Gα proteins. This screening revealed several mutants with reduced biofilm formation, specifically in the presence of *P. aeruginosa*. One of these mutants, Δ*gpaB* (*gpaB* encodes a Gα protein) controls the production of a few SMs shown as important for the *A. fumigatus-P. aeruginosa* biofilm interaction. Pyripyropene A, an SM previously shown as a potent inhibitor of mammalian acyl-CoA cholesterol acyltransferase (19–21), was identified as important for mediating the *A. fumigatus-P. aeruginosa* interaction. The null mutant of *A. fumigatus pyr2* (Δ*pyr2*), encoding a non-reducing polyketide synthase essential for pyripyropene biosynthesis, showed reduced Δ*pyr2* growth during *A. fumigatus-P. aeruginosa* dual biofilm formation and attenuated virulence in a chemotherapeutic murine model of invasive pulmonary aspergillosis (IPA). Our results suggest that *A. fumigatus* pyripyropene is a crucial transkingdom effector, mediating the interaction between different organisms, such as bacteria and mammalian cells.

## RESULTS

### Screening of *A. fumigatus* GPCR, Gα protein, and receptor mutants involved in the *A. fumigatus-P. aeruginosa* biofilm formation

**Fig 1:**
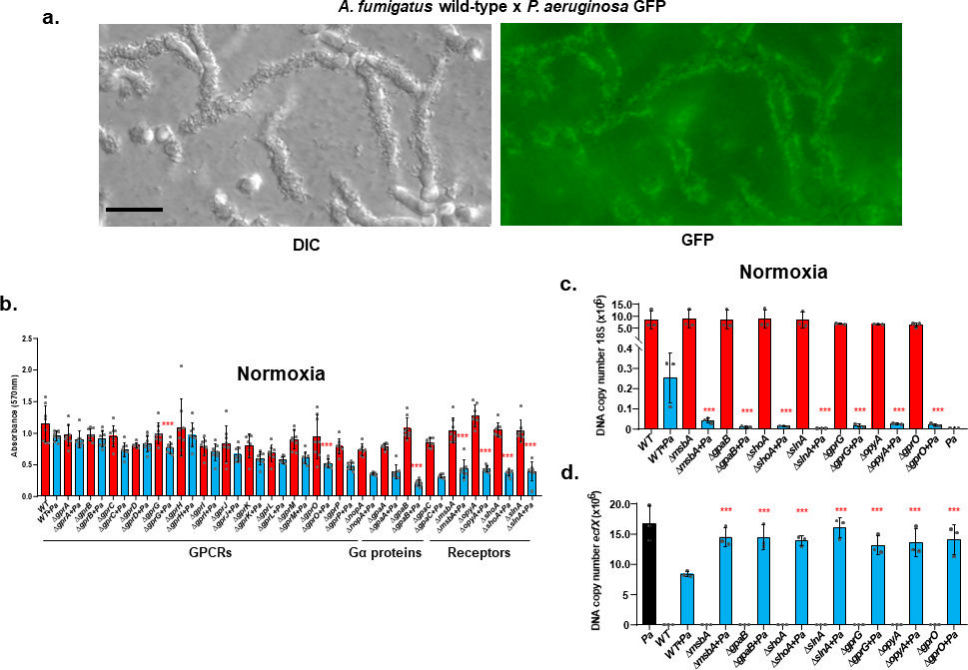
Identification of *A. fumigatus* mutants that have reduced dual biofilm formation with *P. aeruginosa*. (a) *A. fumigatus* conidia were grown for 12 h at 37°C, and *P. aeruginosa* green fluorescent protein (GFP) was added to the culture medium, and both were allowed to grow for 2 h more at 37°C. Bar, 5 µm. (b) The *A. fumigatus* wild-type and mutant strains were incubated for 48 h at 37°C in normoxia in the presence and absence of *P. aeruginosa* (+*Pa*), and biofilm formation was assessed with crystal violet. The results are the average of eight repetitions ± standard deviation. ***, *P* < 0.001. Statistical analysis was performed by using the Dunnett’s multiple comparison test. The biofilm formation of the mutant alone was compared to the wild-type, and when they do not show statistical differences, the dual biofilm formation of the mutant was compared with the dual biofilm formation of the wild-type. (c and d) The wild-type and selected mutants from panel b were incubated or not in the presence of *P. aeruginosa* for 5 days at 37°C. qPCR results for *A. fumigatus* 18S and *P. aeruginosa ecfX* DNA. (b–d) Red bars, *A. fumigatus* wild-type and mutants biofilm formation alone; blue bars, *A. fumigatus* wild-type and mutants dual biofilm formation with *P. aeruginosa*; black bar, *P. aeruginosa* biofilm formation alone. Statistical analysis was performed by using the Dunnett’s multiple comparison test. The dual biofilm formation of *P. aeruginosa* and *A. fumigatus* mutants was compared with the dual biofilm formation of the *P. aeruginosa* and *A. fumigatus* wild-type. The results are the average of three repetitions ± standard deviations. ***, *P* < 0.001.

The initial steps of the interaction between *A. fumigatus* and *P. aeruginosa* are mediated by the adhesion of bacteria to the hyphal surface (Fig. 1a). Hypothesizing that fungal receptors could mediate this bacterial interaction, we screened a collection of null mutants for 14 GPCRs, 3 Gα proteins (*gpaA-B*), 3 MAPK receptors (*msbA*, *opyA*, and *shoA*), and a sensor histidine kinase/response regulator (*slnA*) in both normoxia and hypoxia conditions. Initially, we compared the biofilm formation of these mutants with the corresponding wild-type (WT) and with *P. aeruginosa* by staining with crystal violet (CV). In both normoxia and hypoxia conditions, we observed that Δ*gprG*, Δ*gprO*, Δ*gpaB*, Δ*msbA*, Δ*opyA*, Δ*shoA*, and Δ*slnA* had significantly reduced biofilm formation in the presence of *P. aeruginosa*, but had comparable biofilm formation to the wild-type in its absence (Fig. 1b; Table S1 at https://doi.org/10.6084/m9.figshare.28304309). Since both normoxia and hypoxia conditions provided similar results, we decided to perform the subsequent experiments in normoxia conditions. These results were refined by estimating the *P. aeruginosa* and *A. fumigatus* DNA copy number by quantitative PCR (qPCR) using *P. aeruginosa ecfX* (encoding an extracytoplasmic function sigma factor unique to *P. aeruginosa*, also annotated as *hxuI*) and *A. fumigatus* 18S DNA (22–24). Corroborating the biofilm biomass results, *A. fumigatus* DNA copy number of the mutants decreased in the presence of *P. aeruginosa* compared to the wild-type (Fig. 1c). In contrast, we detected a higher *P. aeruginosa* DNA copy number during biofilm formation with the *A. fumigatus* mutants than with the wild-type strain (Fig. 1d). The reduced growth of *A. fumigatus* mutants appeared to be specific for dual biofilm interaction since dual planktonic growth of the same mutants showed similar DNA copy numbers of both *A. fumigatus* and *P. aeruginosa* (Fig. S1a at https://doi.org/10.6084/m9.figshare.28304309). These results suggest that *A. fumigatus-*identified mutants have a lower ability to interact with and establish a dual *A. fumigatus* × *P. aeruginosa* (WT×Pa) biofilm than the wild-type strain.

### Transcriptional profiling for the *A. fumigatus* Δ*gpaB* mutant interaction with *P. aeruginosa* during early dual biofilm cultures

**Fig 2:**
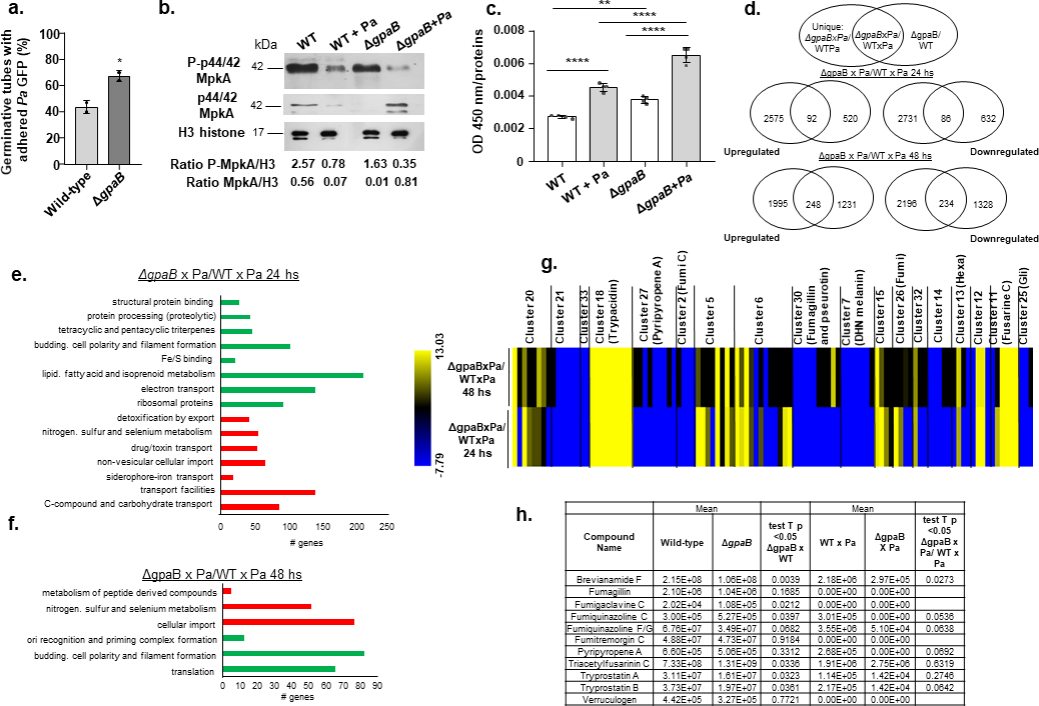
Pyripyropene production is dependent on GpaB, and it is not produced in the Δ*gpaB* mutant under dual biofilm formation of *A. fumigatus* with *P. aeruginosa*. (a) The percentage of *Af* wild-type and Δ*gpaB* mutant germlings that have adhered *P. aeruginosa* labeled with green fluorescent protein (Pa GFP) to their surface. These results are the average of two independent experiments ± standard deviation. In each experiment, 100 germlings were evaluated. Statistical analysis was performed by using *t*-test. (b) Western blot analysis for MpkA and P-MpkA of the wild-type and Δ*gpaB* biofilm formation (96 h at 37°C) in the presence and absence of *P. aeruginosa*. (c) PKA activity of the wild-type and Δ*gpaB* biofilm formation (96 h at 37°C) in the presence and absence of *P. aeruginosa*. The results are the average of three independent biological repetitions and are expressed as average ± standard deviation. Statistical analysis was performed by using Tukey’s multiple comparison test, **, *P* < 0.0015 and ****, *P* < 0.0001. (d) Transcriptional profiling for the *A. fumigatus* Δ*gpaB* mutant interaction with *P. aeruginosa* during early dual biofilm cultures. Venn diagram comparing the genes specifically expressed in the Δ*gpaB* mutant relative to the wild-type strain during *A. fumigatus-P. aeruginosa* dual biofilm formation. Unique expressed genes refer to genes that are expressed only during the dual biofilm interaction but not during *A. fumigatus* wild-type and Δ*gpaB* biofilm formation. (e and f) A summary of the FunCat terms over-represented upregulated (red color) or downregulated (green color) (adjusted *P*-value <0.05) in log2FC ΔgpaB×Pa/WT×Pa at 24 and 48 h. (g) Heat map of the differentially expressed genes encoding proteins involved in the production of secondary metabolites in ΔgpaB×Pa/WT×Pa at 24 and 48 h. (h) Relative secreted metabolites produced by *A. fumigatus* wild-type and Δ*gpaB* in the absence and presence of *P. aeruginosa* during biofilm formation.

Due to the central role played by Gα proteins in signal transduction regulation, we chose Δ*gpaB* as an initial step to understand which pathways are controlled by GpaB that mediate WT×Pa interactions during early dual biofilm cultures. Interestingly, there are more germlings with adhered *P. aeruginosa* in the Δ*gpaB* than in the wild-type strain (Fig. 2a), suggesting GpaB can modulate the number of *P. aeruginosa* that can adhere to the germlings. We have previously demonstrated that *A. fumigatus* Gα proteins interact with GPCRs and modulate the phosphorylation of the MAPK MpkA and protein kinase A (PKA) activity (25, 26). There is a reduction of at least twofold in total and phosphorylated MpkA in the *A. fumigatus* wild-type and Δ*gpaB* mutant biofilm formation alone when compared to either *A. fumigatus* WT or Δ*gpaB* co-culture with *P. aeruginosa* (Pa) (WT×Pa and ΔgpaB×Pa) (Fig. 2b). However, this reduction is at least twofold lower when the P-MpkA/H3 ratio in ΔgpaB×Pa is compared to WT×Pa biofilm formation. PKA activity is higher in the Δ*gpaB* mutant than in the wild-type in the absence or presence of *P. aeruginosa* (Fig. 2c). There is a significant increase of about 50% in the PKA activity upon dual biofilm formation in the WT×Pa 96 h interaction when compared to the WT biofilm formation alone (Fig. 2c). The ΔgpaB×Pa PKA activity is about 70% higher than Δ*gpaB* biofilm formation alone (Fig. 2c).

As an initial step to understand which genes are controlled by GpaB, we performed RNAseq transcriptional profiling and concentrated our attention only on *A. fumigatus* RNA expression. Total RNA was extracted from early ΔgpaB×Pa dual interactions at 24 and 48 h and compared to the corresponding *A. fumigatus* wild-type WT×Pa dual interactions. Differentially expressed genes (DEGs) were defined as those with a minimum of a twofold change in gene expression (log2FC ≥ 1.0 and ≤ −1.0; false discovery rate (FDR) of 0.05; Tables S2 and S3 at https://doi.org/10.6084/m9.figshare.28304309) when compared early ΔgpaB×Pa biofilm formation at 24 and 48 h with ΔgpaB×WT biofilm formation at 24 and 48 h. This analysis allowed us to identify genes that could be modulated during biofilm formation and are not related to the dual interaction. We observed 2,575 and 1,995 unique genes upregulated and 2,731 and 2,196 downregulated at 24 and 48 h, respectively (Fig. 2d).

FunCat enrichment analyses showed that ΔgpaB×Pa at 24 h has a decreased expression (relative to WT×Pa) of genes encoding ribosomal proteins, electron transport, lipid, fatty acid and isoprenoid metabolism, budding, cell polarity, and filament formation and an increased expression of genes encoding transporters, C-compound and carbohydrate metabolism, nitrogen, sulfur, and selenium metabolism (Fig. 2e). At 48 h, ΔgpaB×Pa downregulated genes were enriched for functions involved in translation, budding, and filament formation; upregulated genes were enriched for functions involved in cellular import, nitrogen, sulfur, and selenium metabolism (Fig. 2f).

The genes that encode SMs are generally organized in biosynthetic gene clusters (BGCs) (27), and *A. fumigatus* has at least 598 SM-associated genes distributed among 33 BGCs (28, 29). Previously, we detected eight SMs (demethoxyfumitremorgin C, fumitremorgin, ferrichrome, ferricrocin, triacetylfusigen, gliotoxin, gliotoxin E, and pyripyropene A) secreted by *A. fumigatus* during biofilm formation in coculture with *P. aeruginosa* (18). Here, we observed three genes (GliZ, GliI, and GliT) in the gliotoxin BGC (cluster 25) and all the genes in the pyripyropene A BGC (cluster 27) as downregulated in the ΔgpaB×Pa (Fig. 2g). In addition to these two clusters, clusters 21, 2 (fumigaclavine C), 30 (fumagillin and pseurotin), and 7 (dihydroxynaphthalene melanin [DHN-melanin]) were downregulated while clusters 18 (trypacidin) and 11 (fusarin C) were upregulated in the ΔgpaB×WT (Fig. 2g).

We also evaluated the SM production by using high-performance liquid chromatography coupled with high-resolution tandem mass spectrometry (LC-HRMS/MS) to identify SMs in the *A. fumigatus* wild-type, Δ*gpaB*, and *P. aeruginosa* biofilm supernatants (Fig. 2h and Table S4 at https://doi.org/10.6084/m9.figshare.28304309). SMs were identified either based on MS/MS spectral matching with the Global Natural Product Social Molecular Networking (GNPS) public libraries (30) or through fragment fingerprint prediction and comparison with chemical structure libraries (31, 32), considered level 2 and 3 identifications, respectively (33) (Table S4 at https://doi.org/10.6084/m9.figshare.28304309). In the presence of *P. aeruginosa*, *A. fumigatus* Δ*gpaB* was not able to produce fumiquinazoline C and pyripyropene, while *A. fumigatus* Δ*gpaB* has a comparable production of both metabolites to the wild-type strain when grown in isolation (Fig. 2h). In the presence of *A. fumigatus* Δ*gpaB*, *P. aeruginosa* has a significant decreased or increased production of 2-(non-1-enyl)-4-hydroxyquinoline N-oxide, 2-(non-1-enyl)quinolin-4-ol, 2-(undec-1-enyl)quinolin-4-ol, phenazine-1-carboxamide, pyochelin, pyochelin methyl ester, and pyocyanin when compared to WT × Pa (Table S4 at https://doi.org/10.6084/m9.figshare.28304309).

These results suggest that *A. fumigatus* GpaB is important for growth in dual biofilms with *P. aeruginosa*, modulating the MAPK MpkA phosphorylation and PKA activity, the transcription of genes encoding BGC proteins, and affecting the production of pyripyropene and fumiquinazoline.

### Pyripyropene production in dual biofilm culture is dependent on Δ*gpaB* and is important for *A. fumigatus-P. aeruginosa* interactions

**Fig 3:**
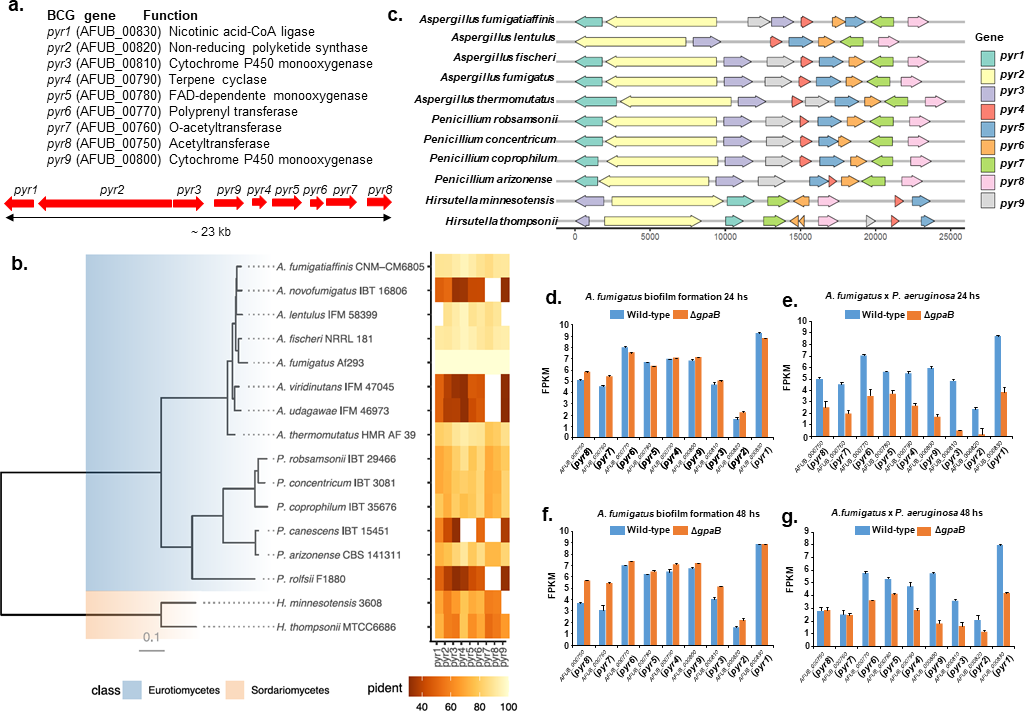
Pyripyropene production is dependent on GpaB during *A. fumigatus-P. aeruginosa* dual biofilm formation. (a) Pyripyropene BGC genes and chromosomal organization. (b) Phylogenetic distribution of the pyripyropene cluster. (c) Organization of the pyripyropene BGC of several different species. (d–g) FPKM (fragments per kilobase of transcript per million fragments mapped) values from the nine genes of pyripyropene BGC, obtained from the RNAseq during *A. fumigatus* biofilm formation, comparing the WT and Δ*gpaB* for *A. fumigatus* alone and in the presence of *P. aeruginosa*. Treatments include biofilm formation at 24 and 48 h.

We have previously shown that pyripyropene is produced by *A. fumigatus* during biofilm formation in the presence of *P. aeruginosa* (18). Pyripyropenes are potent inhibitors of mammalian acyl-CoA:cholesterol acyltransferase (19). However, the ecological function of pyripyropene needs to be clarified, as does how it affects the *A. fumigatus-P. aeruginosa* dual interaction during biofilm formation. The pyripyropene BGC is composed of nine genes and has a narrow interspecies distribution among filamentous fungi (Fig. 3a through c). We identified the pyripyropene BGC in *A. fumigatus* and putatively in the genomes of 10 additional species (Fig. 3b). The phylogeny of those species, as well as of a few additional closely related species, was inferred based on 608 BUSCO genes present in all of them. The BGC is found in species of the closely related genera *Aspergillus* (in five species) and *Penicillium* (four species) (class Eurotiomycetes) and the distantly related *Hirsutella* (two species) (class Sordariomycetes) (Fig. 3b and c). The nematode endoparasitic fungus *Hirsutella minnesotensis* and the entomopathogenic fungus *Hirsutella thompsonii* (Ophiocordycipitaceae [Hypocreales, Ascomycota]) are the most phylogenetically distant species from *A. fumigatus* (34, 35).

The expression of *pyr* BGC genes is comparable between the wild-type and Δ*gpaB* strains during *A. fumigatus* biofilm formation at 24 and 48 h, except for *pyr8* (AFUB_00750) and *pyr7* (AFUB_00760) that have increased expression in the Δ*gpaB* mutant at 48 h biofilm formation (Fig. 3d and f). However, these nine genes have substantial decreased expression in the Δ*gpaB* mutant at 24 and 48 h biofilm dual interaction with *P. aeruginosa* (except again for *pyr8* and *pyr7* that have comparable expression with the wild-type strain at 48 h dual biofilm interaction; Fig. 3e and g). These results suggest GpaB is important for the transcriptional modulation of the genes involved in pyripyropene production during coculture with *P. aeruginosa*.

**Fig 4:**
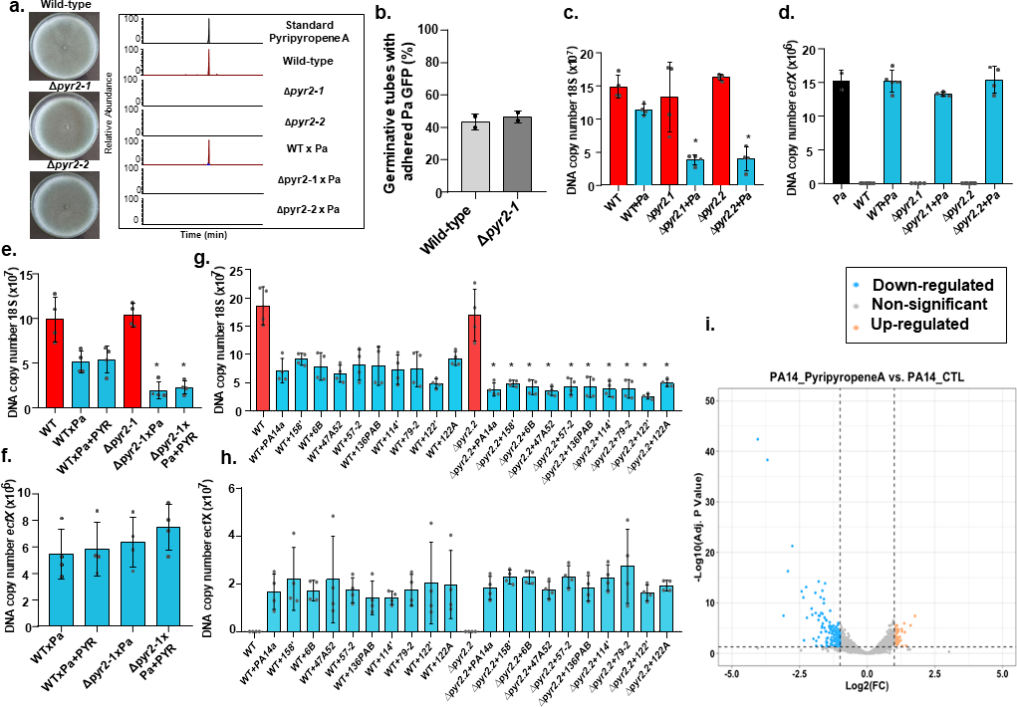
Pyripyropene production is important for *A. fumigatus-P. aeruginosa* dual biofilm formation. (a) *A. fumigatus* wild-type, Δ*pyr2-1*, and Δ*pyr2-2* strains were grown for 5 days at 37°C. Chromatograms of pyripyropene production in the wild-type, Δ*pyr2-1*, and Δ*pyr2-2* strains after 5 days at 37°C biofilm formation and dual Af×Pa, Δpyr2-1×Pa, and Δpyr2-2×Pa dual biofilm formation. (b) The percentage of germlings that have adhered *P. aeruginosa* labeled with green fluorescent protein (Pa GFP) to their surface. These results are the average of two independent experiments ± standard deviation. In each experiment, 100 germlings were evaluated. Statistical analysis was performed by using *t*-test. (c–f) *P. aeruginosa* and *A. fumigatus* wild-type and mutants grown for 5 days at 37°C. qPCR results for *A. fumigatus* 18S and *P. aeruginosa ecfX* DNA. Pyripyropene (PYR, 1 µg) was added or not to the experiments. The results are the average of three repetitions ± standard deviations. **, *P* < 0.01. (g and h) *P. aeruginosa* clinical isolates and *A. fumigatus* wild-type and mutants grown for 5 days at 37°C. qPCR results for *P. aeruginosa ecfX* and *A. fumigatus* 18S DNA. The results are the averages of three repetitions ± standard deviations. *, *P* < 0.05; **, *P* < 0.01. (i) Volcano plot of comparative transcriptomic analysis (RNAseq) from wild-type (WT) PA14 versus PA14 treated with 10 µg/mL pyripyropene A grown in RPMI (RPMI 1640 medium, HEPES) (*n* = 3). Statistically significant values are defined by *P*-values >0.05 and a log2FC outside the thresholds of −1 and 1.

We deleted the *pyr2* gene encoding the non-reducing polyketide synthase involved in the first step of the pyripyropene biosynthesis (Fig. S2a). Two independent deletion strains (Δ*pyr2-1* and Δ*pyr2-2*) have comparable phenotypes of growth and conidiation with the wild-type in different single carbon sources, such as glucose 1%, ergosterol 0.01%, and cholesterol 0.1% in 72 h, except for growth at 72 h in glucose 1%, suggesting *pyr2* mutants are more susceptible to glucose starvation than the wild-type strain (Fig. S2b at https://doi.org/10.6084/m9.figshare.28304309), and none of these mutants were able to produce pyripyropene during *A. fumigatus* biofilm formation or during dual biofilm formation in the presence of *P. aeruginosa* (Fig. 4a). We also have not observed any growth defects in the Δ*pyr2* mutants in the presence of cell wall-damaging agents, such as Congo red and calcofluor white, and oxidative stressing agents, such as menadione (Fig. S2c at https://doi.org/10.6084/m9.figshare.28304309).

There is a comparable number of *A. fumigatus* germlings with adhered *P. aeruginosa* in the Δ*pyr2-1* mutant and in the wild-type strain (Fig. 4b), suggesting that Pyr2 is not directly affecting the adhesion of *P. aeruginosa* to the germlings. We investigated if the absence of *A. fumigatus* pyripyropene production could affect WT×Pa biofilm formation by estimating the *P. aeruginosa* and *A. fumigatus* DNA copy number by qPCR. *A. fumigatus* DNA copy number of the Δ*pyr2* mutants decreased in the presence of *P. aeruginosa* strain without any alteration in the *P. aeruginosa* DNA copy number (Fig. 4c and d). Attempts to restore *A. fumigatus* Δ*pyr2-1* to wild-type levels by adding pyripyropene 1 µg/mL have failed (Fig. 4e and f). Dual planktonic growth of Δ*pyr2* mutants with *P. aeruginosa* showed similar DNA copy numbers to *A. fumigatus* wild-type-*P. aeruginosa* planktonic cultures (Fig. S1b at https://doi.org/10.6084/m9.figshare.28304309). We also tested if the lack of pyripyropene could affect the *A. fumigatus* biofilm formation in the presence of *P. aeruginosa* mucoid and non-mucoid isolates from CF patients (36). The Δ*pyr2-1* mutant showed decreased growth with all the nine clinical isolates, and there was no increase in DNA copy number in the *P. aeruginosa* clinical isolates during dual biofilm formation (Fig. 4g and h).

To assess whether pyripyropene affects *P. aeruginosa* growth or physiology, we cultured the *P. aeruginosa* strains PA14 and PAO1 with varying concentrations of pyripyropene (0 µg/mL–20 µg/mL). Across all tested concentrations, no significant impact on *P. aeruginosa* growth was observed, suggesting pyripyropene is not acting as an inhibitor or a quorum sensing molecule (Fig. S3 at https://doi.org/10.6084/m9.figshare.28304309). This indicates that the observed alterations in the Δ*pyr2* mutant during coculture with *P. aeruginosa* are not attributable to changes in bacterial growth in the absence of pyripyropene production.

Although pyripyropene did not directly affect *P. aeruginosa* growth in our assays, it may influence its physiology, potentially altering its competitiveness within coculture biofilms with *A. fumigatus*. To explore this, we exposed *P. aeruginosa* to pyripyropene (10 µg/mL) for 3 h, alongside a solvent control, during exponential growth in RPMI (RPMI 1640 medium, HEPES). We then extracted total RNA to specifically assess how pyripyropene impacts *P. aeruginosa* gene expression. Differentially expressed genes were defined as those showing at least a twofold change in expression (log2FC ≥ 1.0 or ≤ −1.0; FDR ≤ 0.05; Table S5 at https://doi.org/10.6084/m9.figshare.28304309). Our analysis revealed 47 genes upregulated and 154 genes downregulated in response to pyripyropene exposure (Fig. 4i).

Two genes within the phenazine biosynthesis operon, *phzA2* and *phzB2*, were upregulated by 1.6-fold and 1.76-fold, respectively. Additionally, genes encoding subunits of the *P. aeruginosa* ATP synthase complex (*atpB*, *atpF*, *atpH*, *atpG*, and *atpD*) exhibited approximately twofold upregulation in response to pyripyropene exposure. However, previously, we have not observed significant growth differences between dual biofilm formation between the *P. aeruginosa* wild-type, Δ*phzA1,* and Δ*phzA2* mutants and *A. fumigatus* wild-type (18). In contrast, genes downregulated in *P. aeruginosa* following pyripyropene exposure included those involved in acetate/ethanol oxidation (*eraR*, *eraS*, *ercS*, *adhA*, and *acsA*), ferrous iron transport (*feoA*, *feoB*), and an uncharacterized operon (*PA14_15120–PA14_15130*) (Table S5 at https://doi.org/10.6084/m9.figshare.28304309). Despite the relatively high concentration of pyripyropene used (10 µg/mL), the modest fold changes suggest that pyripyropene does not induce broad alterations in *P. aeruginosa* physiology or its transcriptome. This implies that the loss of pyripyropene production in the Δ*pyr*2 mutant may have a more pronounced effect on *A. fumigatus* within dual biofilms than on *P. aeruginosa*.

**Fig 5:**
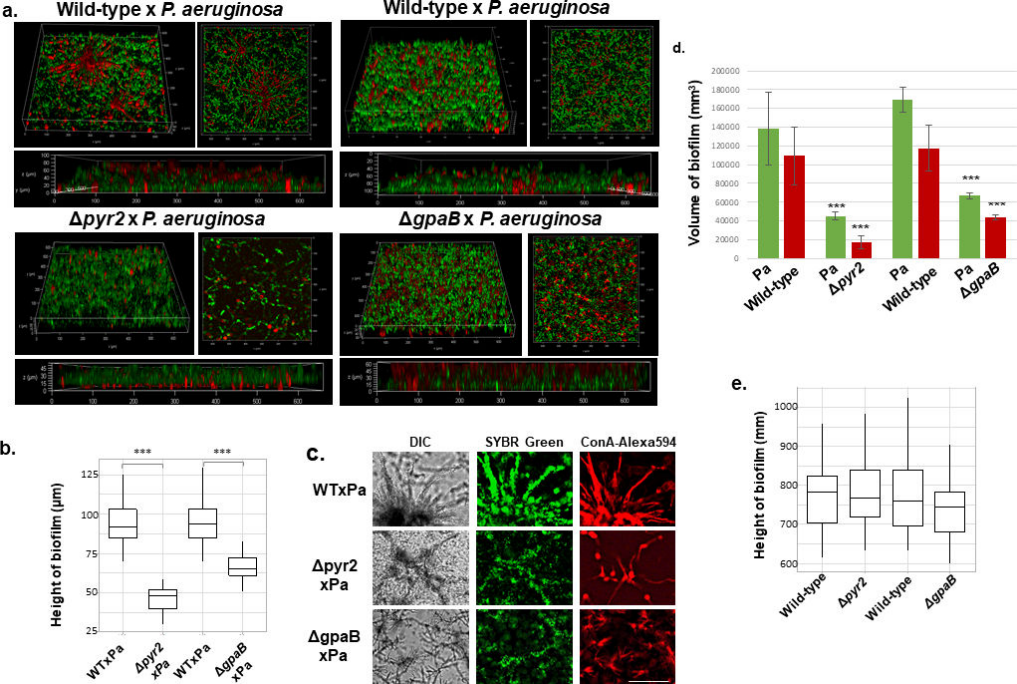
Confocal microscopy shows that *A. fumigatus* Δ*gpaB* and Δ*pyr2* have reduced dual biofilm formation in the presence of *P. aeruginosa*. (a) Dual biofilm formation between *A. fumigatus* wild-type, Δ*gpaB* and Δ*pyr2*, and *P. aeruginosa*. (b) Analysis of the biofilm height (µm) of the dual biofilm formation of *A. fumigatus* wild-type, Δ*gpaB* and Δ*pyr2*, and *P. aeruginosa*. The results are the average of 18 independent images ± standard deviation. Statistical analysis was performed by using *t*-test. ***, *P* < 0.001. (c) Selected images of WT×Pa, ΔgpaB×Pa, and Δpyr2×Pa dual biofilm formation. (d) Each volume of Pa and Af biofilm was measured by Volocity software from the fluorescent signal of 3D images. 325 × 325 µm^2^, four positions, respectively. The results are the average of four positions ± standard deviations. ***, *P* < 0.001. (e) Analysis of the biofilm height (µm) of the biofilm formation of *A. fumigatus* wild-type, Δ*gpaB,* and Δ*pyr2*. The results are the average of 18 independent images ± standard deviation. Statistical analysis was performed by using *t*-test. n.s., not significant.

We applied fluorescent microscopy aiming to validate if there was a reduced biofilm formation in both Δ*gpaB* and Δ*pyr2* mutants. Dual biofilm formation was established between the wild-type, Δ*gpaB*, and Δ*pyr2* mutant strains (stained with concanavalin A-Alexa 594) and *P. aeruginosa* (stained with SYBR Green) (Fig. 5a, b and c). There was a reduction in *A. fumigatus* growth within ΔgpaB×Pa and Δpyr2×Pa dual biofilms (Fig. 5a and c), and a reduction of about 40 and 60% in the biofilm height was observed for ΔgpaB×Pa and Δpyr2×Pa, respectively, when compared to Afwt×Pa dual biofilms (Fig. 5b; Table S6; Movies S1 to S3 at https://doi.org/10.6084/m9.figshare.28304309). In contrast, when growing without *P. aeruginosa,* Δ*gpaB* and Δ*pyr2* have a comparable biofilm formation to the wild-type strain (Fig. 5d and e; Fig. S4; Table S5 at https://doi.org/10.6084/m9.figshare.28304309).

Taken together, these results suggest that GpaB is essential for pyripyropene formation within dual biofilms, and pyripyropene is partially required for *A. fumigatus* growth during dual biofilm formation with *P. aeruginosa*.

### Pyripyropene affects *A. fumigatus* lipid metabolism

Pyripyropene A is an SM previously shown as a potent inhibitor of mammalian acyl-CoA cholesterol acyltransferase (ACATS) (19–21). The reduced dual biofilm growth of *A. fumigatus* Δ*pyr2* in the presence of *P. aeruginosa* could be explained by two hypotheses: (i) pyripyropene is affecting a target in the bacterial cell or (ii) the lack of pyripyropene is affecting biofilm establishment specifically in the presence of *P. aeruginosa* since Δ*pyr2* alone has a comparable biofilm formation to the wild-type strain alone (Fig. 4b, 5d and e). This second hypothesis opens the possibility that pyripyropene is affecting the fungal metabolism only in the presence of *P. aeruginosa*, providing the fungus with a competitive and fitness advantage. However, the minimal inhibitory concentration (MIC) of *P. aeruginosa* pyripyropene is >10 µg/mL (determined in RPMI medium), which is the same as the *A. fumigatus* MIC (determined in minimal medium [MM] + 1% glucose and MM + 1% ergosterol); these data do not support the hypothesis that *P. aeruginosa* growth is directly impacted by pyripyropene in dual biofilms.

**Fig 6:**
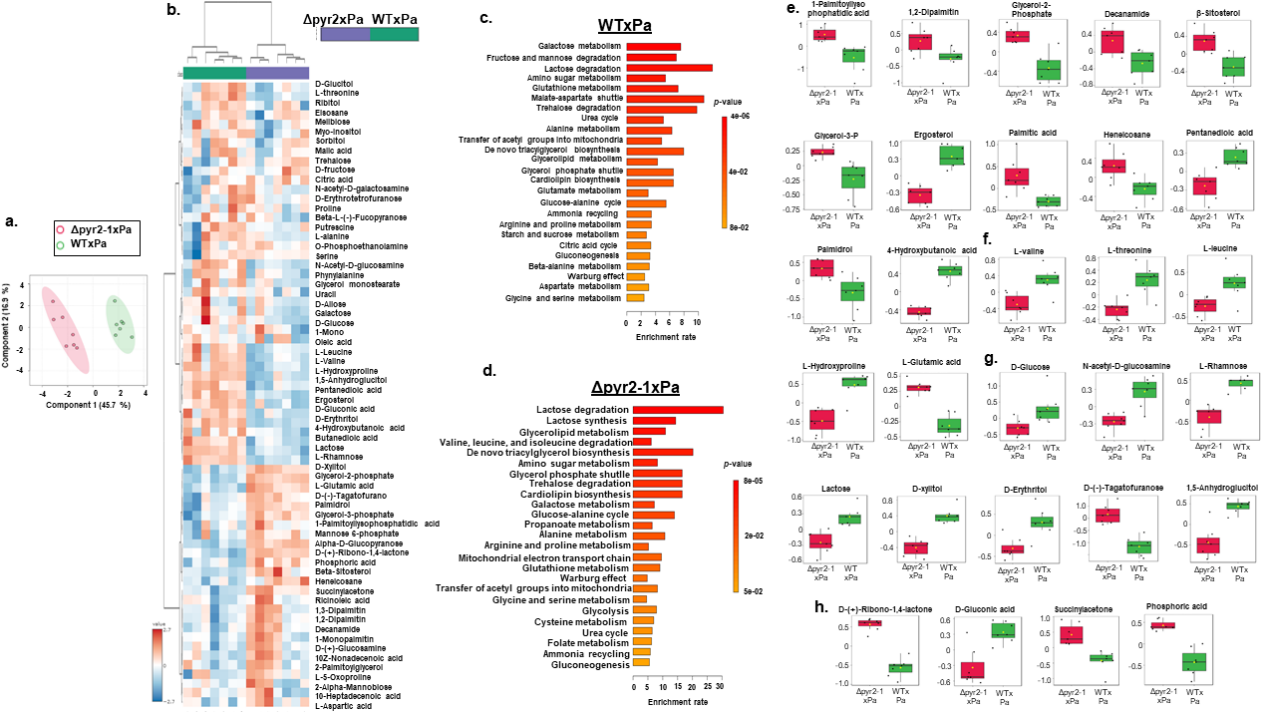
GC-MS analysis of the dual biofilm interaction *A. fumigatus* wild-type and *pyr2* with *P. aeruginosa*. (a) Principal component analysis distribution of nine repetitions for WT×Pa and Δpyr2-1×Pa. Partial least squares (PLS) model. Validation parameters [*R*^2^(*X*) = 0.98, *Q*^2^ = 0.95]. (b) Heat map of the metabolites extracted from seven repetitions of WT×Pa and Δpyr2-1×Pa. (c and d) Enrichment analysis by MetaboAnalyst (https://www.metaboanalyst.ca/) of the WT×Pa and Δpyr2×Pa. The enrichment ratio is computed by hits/expected, where hits = observed hits and expected = expected hits. (e) Lipids, (f) amino acids, (g) carbohydrates, and (h) miscellaneous. Box plots for selected metabolites of WT×Pa and Δpyr2-1×Pa. Results are the average of seven repetions ± standard deviation. Statistical analysis was performed with one-way analysis of variance, followed by Tukey’s multiple comparison test.

We applied gas chromatography-mass spectrometry (GC-MS) analysis to identify the metabolites present in the WT×Pa and Δpyr2-1×Pa dual biofilm interactions. There was a clear distinction between the Δpyr2-1×Pa and WT×Pa metabolisms (Fig. 6a through 6g; Tables S7 and S8 at https://doi.org/10.6084/m9.figshare.28304309). MetaboAnalyst-based analysis (https://www.metaboanalyst.ca/) of the WT×Pa and ΔpyrA×Pa showed enrichment for amino acid, lipid, and carbohydrate metabolism in both interactions (Fig. 6b through d). In the Δpyr2-1×Pa interaction, we observed an increased accumulation of several lipids or lipid precursors, such as 1-palmitoyl lysophosphatidic acid, 1,2-dipalmitin, glycerol-2-phosphate, decanamide, palmitic acid, heneicosane, β-sitosterol, and palmidrol; in contrast, there is an increased accumulation of ergosterol, 4-hydroxybutanoic acid, and pentanedioic acid in the WT×Pa interaction (Fig. 6e). The β-sitosterol has already been reported as produced by *Aspergillus niger* (37, 38). To our knowledge, there are no reports about β-sitosterol production by *P. aeruginosa*. There is also more accumulation of amino acids such as L-valine, L-threonine, L-leucine, and L-hydroxyproline in the WT×Pa interaction but accumulation of L-glutamic acid in the Δpyr2×Pa interaction (Fig. 6f). L-rhamnose and D-gluconic acid were also identified as less produced by Δpyr2-1×Pa than WT×Pa (Fig. 6f and h). *P. aeruginosa* can produce D-gluconic acid (39), and *Pseudomonas* spp. can produce D-gluconic acid as an effective antifungal compound against *Gaeumannomyces graminis*, the agent of the take-all disease of wheat (40). Rhamnose is the precursor of *P. aeruginosa* rhamnolipids, which have been shown to modulate *A. fumigatus* galactosaminogalactan and melanin production, hyphal wall thickness, and cell wall chitin concentrations through the inhibition of β-1,3 glucan synthase (12, 13). There is a reduced accumulation of N-acetyl-D-glucosamine in the mutant strain, suggesting that there is a reduction of chitin accumulation in the mutant cell walls (Fig. 6g).

Collectively, these findings indicate that lipids undergo more contrasting modifications during the interaction between Δpyr2-1×Pa and WT×Pa. Additionally, the mutant strain exhibits more significant modulation of other chemicals that play a crucial role in establishing the connection compared to the wild-type strain.

### Pyripyropene and *A. fumigatus* ergosterol metabolism

Sterols are present in two forms in *Saccharomyces cerevisiae*: (i) as free sterols located mainly in the plasma membrane and as (ii) sterol esters sequestered in cytosolic lipid particles called lipid droplets (LDs) (41–44). *S. cerevisiae* Are1p and Are2p (that exhibit 49% identity to each other and 20% identity to human ACATS) are acyl-CoA:sterol acyltransferases that catalyze the sterol esterification (41–44). To investigate the hypothesis that pyripyropene could modulate ergosterol sterification in *A. fumigatus*, we first tested the interaction between pyripyropene and drugs that target ergosterol and sphingolipid biosynthesis. The synergy scores for the interaction between pyripyropene and voriconazole and pyripyropene and cerulenin are 0.37 and −0.601 (both additive interactions) when grown in MM + glucose and 7.61 (additive interaction) and 10.4 (synergism interaction) when grown in MM + ergosterol (Fig. S5 at https://doi.org/10.6084/m9.figshare.28304309).

**Fig 7:**
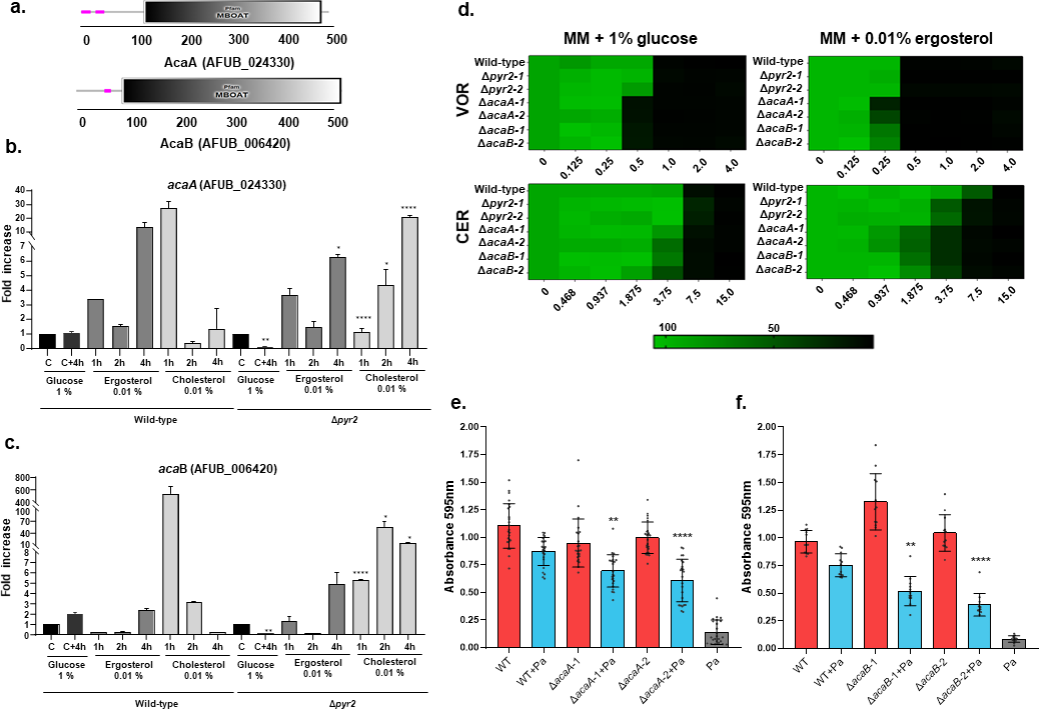
Pyripyropene is affecting the ergosterol metabolism in *A. fumigatus*. (a) Organization of the *A. fumigatus* AcaA and AcaB. Both encoding proteins share MBOAT domains from a family of membrane proteins that contain a variety of acyltransferase enzymes (Pfam accession number PF03062, available in https://www.ebi.ac.uk/interpro/entry/pfam/PF03062/). (b and c) RTqPCR analysis for *acaA* and *acaB. A. fumigatus* wild-type and Δ*pyr2* were grown in MM + 1% glucose for 16 h at 37°C (C = control) and transferred to either MM + glucose 1% (C + 4 h), MM + ergosterol 0.01%, or MM + cholesterol 0.01%. Results are the average of three independent experiments ± standard deviation. Statistical analysis was performed with one-way analysis of variance, followed by Tukey’s multiple comparison test. *P < 0.05, **P < 0.01, ****P < 0.0001. (d) Alamar blue assays for *A. fumigatus* strains grown in liquid MM + 1% glucose or 0.01% ergosterol for 48 h at 37°C with the indicated concentrations of voriconazole or cerulenin. (e and f) The wild-type and *acaA* and *acaB* null mutants were incubated or not in the presence of *P. aeruginosa* for 5 days at 37°C. qPCR results for *A. fumigatus* 18S and *P. aeruginosa efcX* DNA. Statistical analysis was performed by using the Dunnett’s multiple comparison test. The dual biofilm formation of *P. aeruginosa* and *A. fumigatus* mutants was compared with the dual biofilm formation of the *P. aeruginosa* and *A. fumigatus* wild-type. The results are the average of six repetitions ± standard deviations. ***, *P* < 0.001.

Secondly, we characterized the *A. fumigatus* ACAT homologs that esterify ergosterol and are possible targets for activity modulation by pyripyropene. *A. fumigatus* has two homologs for acyl-CoA cholesterol acyltransferases, AFUB_006420 and AFUB_024330 (here called AcaA and AcaB, respectively [Fig. 7a]). Both encoding proteins share MBOAT domains (for membrane-bound O-acyltransferase; [45]) from a family of membrane proteins that contain a variety of acyltransferase enzymes (Pfam accession number PF03062; Fig. 7a). AcaA and AcaB are the *S. cerevisiae* Are1p and Are2p sterol acyltransferase homologs (4e − 60 and 30% identity for Are1p and 8e − 80 and 36% identity for Are2p, respectively). The *acaA* gene is not differentially expressed during WT×Pa and ΔgpaB×Pa dual interactions, while *acaB* is upregulated (1.341) at WT×Pa 48 h interaction and downregulated at ΔgpaB×Pa 24 h dual interactions, respectively (Tables S2 and S3 at https://doi.org/10.6084/m9.figshare.28304309). When the *A. fumigatus* wild-type and Δ*pyr2* mutant were grown for 16 h in liquid MM + glucose 1% and transferred to MM 1%, ergosterol 0.01%, or cholesterol 0.01%, *acaA* and *acaB* in the Δ*pyr2* mutant showed reduced and delayed mRNA accumulation in all conditions when compared to the wild-type strain (Fig. 7b and c).

The Δ*acaA* and Δ*acaB* mutants presented similar growth rates compared to wild-type *A. fumigatus* in MM supplemented either with glucose, cholesterol, or ergosterol as single carbon sources both in normoxia and hypoxia (Fig. S2c and d and S6 at https://doi.org/10.6084/m9.figshare.28304309). Since pyripyropene and acyl-CoA cholesterol acyltransferases are necessary for ergosterol metabolism, we tested the influence of lipid biosynthesis inhibitors on these mutants growing either in glucose or ergosterol as single carbon sources. The Δ*acaA* and Δ*acaB* mutants were more susceptible to voriconazole (which inhibits the *A. fumigatus* sterol 14-alpha demethylase encoded by *cyp51A*) and cerulenin (an antifungal agent whose activity interferes with or otherwise acts to prevent the formation of fatty acids and sterols; [46]) when grown in MM + 1% glucose or 0.01% ergosterol as single carbon sources (Fig. 7d). The Δ*pyr2* mutants are more susceptible to cerulenin when grown in MM + 0.01% ergosterol as a single carbon source (Fig. 7d). Dual biofilm formation was established between the wild-type, Δ*acaA*, and Δ*acaB* mutant strains (Fig. 7e and f). Consistently, as Δpyr2×Pa, there was also a reduction in biofilm formation of about 50 to 75% in ΔacaA×Pa and ΔacaB×Pa when compared to WT×Pa (Fig. 7e). Interestingly, comparable to the interaction between Δpyr2×Pa, there is no alteration in the *P. aeruginosa* DNA copy number (Fig. 7f).

In summary, these results suggest pyripyropene is affecting the ergosterol metabolism in *A. fumigatus*, possibly through the modulation of AcaA and AcaB acyl-CoA:sterol acyltransferase activities.

### Pyripyropene is important for cytokine production and killing by bone marrow-derived macrophages and virulence

**Fig 8:**
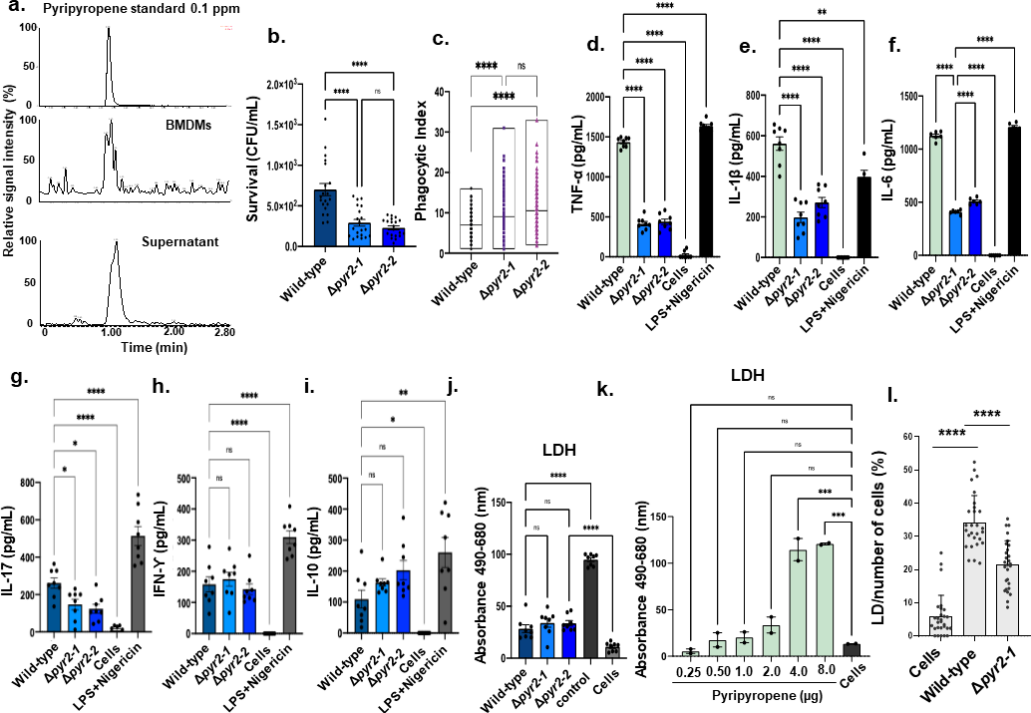
The *A. fumigatus* Δ*pyr2* mutants have reduced viability in the presence of BMDMs and elicited reduced cytokine production. (a) High-performance liquid chromatography (HPLC) chromatograms of three repetitions of the wild-type exposed to BMDMs for 24 h. Supernatants refer to secreted pyripyropene in the presence of BMDMs, while BMDMs refer to pyripyropene that was identified associated with macrophages (either on the surface or intracellularly). (b and c) The *A. fumigatus* Δ*pyr2* mutants have reduced viability and engulfment in the presence of BMDMs when compared to the wild-type strain. (d–i) Pro-inflammatory cytokine production is reduced in the *A. fumigatus* Δ*pyr2* mutants compared to the wild-type strain. (j and k) BMDM viability as measured by LDH release. Positive control is provided by the CyQUANT LDH Cytotoxicity Assay kit (Invitrogen C20300). The results are the average of three independent biological repetitions and are expressed as average ± standard deviation. Statistical analysis was performed using a one-way analysis of variance (Dunnett’s test) for multiple comparisons. ns, not significant, *P < 0.05, **P < 0.01, ***P < 0.001, ****P < 0.0001. (l) LD formation in BMDMs exposed to the wild-type and Δ*pyr2-1* for 24 h at 37°C. Two independent experiments were performed, and 28 microscopy fields with approximately 700 BMDMs in total were counted. Statistical analysis was performed by using Tukey’s multiple comparison test, **, *P*-value < 0.0015, and ****, *P*-value < 0.0001.

To understand the function of the *A. fumigatus* pyripyropene in the establishment of animal infection, we assessed the role of Δ*pyr2* mutants in survival and tissue invasion using *in vitro* models of infection. *A. fumigatus* produces pyripyropene when exposed to bone marrow-derived macrophages (BMDMs), and its presence was observed in both supernatants and macrophages (Fig. 8a). Based on the intensities of the pyripyropene A chromatographic peak in the analyzed standard compound and biological samples, we can estimate that pyripyropene concentration in supernatants and macrophages is below 0.01 µg/mL. The *pyr2* null mutants showed increased killing and phagocytosis by BMDMs compared to the wild-type strain (Fig. 8b and c). We investigated if Pyr2 could play a role in cytokine response specifically of tumor necrosis factor alpha (TNF-α), interleukin-1 beta (IL-1β), IL-6, IL-17, interferon gama (IFN-γ), and IL-10 production in BMDMs (Fig. 8d through i). We observed a significant reduction of all cytokines, except for IL-10 and IFN-γ when Δ*pyr2* mutants were exposed for 24 h to BMDMs (Fig. 8d through i). Δ*pyr2* mutants do not cause more toxicity to BMDMs (as measured by lactate dehydrogenase [LDH] release) when compared to the wild-type strain. In contrast, very high concentrations of pyripyropene can cause more damage to BMDMs (Fig. 8j and k). Cholesteryl esters (CEs) produced by ACATs cannot partition in membranes; they can only coalesce as cytosolic LDs (47). Since pyripyropene can inhibit CE production and affect the number of LDs, we investigated the effect of *pyr2* mutation on LD formation in BMDMs. When the wild-type and Δ*pyr2* mutants are exposed to BMDMs, there are 63% more LDs in the wild-type than in the Δ*pyr2* mutant (Fig. 8l).

**Fig 9:**
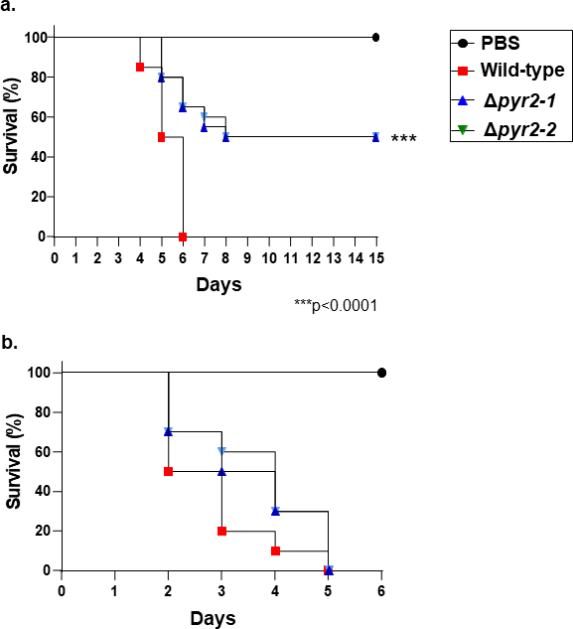
The Δ*pyr2* mutants have attenuated virulence in a murine immunocompetent model of murine IPA. (a) Chemotherapeutic (a) or immunocompetent (b) murine models of IPA. Kaplan-Meier survival curves showing percentages of immunosuppressed (*n* = 10 mice/strain) or immunocompetent (*n* = 5 mice/strain) mice infected intranasally with *A. fumigatus* strains. Phosphate-buffered saline (PBS) was administered in a negative control group (*n* = 10 for each model). The results are the average of two independent experiments with 10 or 5 animals for each treatment. Indicated *P-*values were determined with the use of the Logrank (Mantel-Cox test) and Gehan-Breslow-Wilcoxon test comparing the Δ*pyr2-1* and Δ*pyr2-2* mutants with the wild-type strain, (*, *P* < 0.0001).

Pyripyropene is important not only for *A. fumigatus* dual biofilm interaction with *P. aeruginosa* but also for virulence in animal cells since Δ*pyr2* mutants have decreased virulence in a chemotherapeutic murine model of IPA (Fig. 9a). All mice infected with the *A. fumigatus* wild-type strain died between day 6 post-infection, whereas 50% of the mice infected with the Δ*pyr2* survived for the duration of the experiment (Fig. 9a). Interestingly, the Δ*pyr2* mutants and the wild-type have comparable virulence in the mouse immunocompetent model (Fig. 9b).

These results suggest that *A. fumigatus* pyripyropene is a critical secreted effector important for BMDM phagocytosis and viability, and that pyripyropene can modulate cytokine production and affect LD formation. Pyripyropene is important for dissemination and killing in a murine chemotherapeutic model but not in an immunocompetent model.

## DISCUSSION

*A. fumigatus* and *P. aeruginosa* have close interactions and are ubiquitous constituents of the pulmonary environment in CF patients. Previously, we established an Af×Pa biofilm interaction model that allowed us to investigate the production of SMs from both species (18). Here, we explored in more detail the factors involved in the Af×Pa interaction by isolating several genetic determinants that affect biofilm interaction between *A. fumigatus* and *P. aeruginosa*. We extended this analysis to the *A. fumigatus* transcriptional profiling of early biofilm interaction and screening of *A. fumigatus* mutants of genetic determinants that are possibly involved in either the recognition and/or the response to *P. aeruginosa*. We identified five cell surface receptor (GPCRs GprG and GprO and MAP kinase receptors MsbA, OpyA, and ShoA) and two signal transduction (Gα protein GpaB and the sensor histidine kinase/response regulator SlnA) mutants that have reduced growth during the dual biofilm formation with *P. aeruginosa*. We concentrated our attention on the characterization of the role played by GpaB due to its centrality as a hub in the activation of several signal transduction pathways in *A. fumigatus* (for a review, see reference 48). However, it remains to be investigated if GprG and GprO are interacting with GpaB and which downstream targets are modulated by GPCRs, SlnA, and MAPK receptors.

Fungi recognize the immediate environment and activate their metabolism and development by using receptors that sense metabolites and physicochemical changes (like pH and temperature). GPCRs are the large class of receptors generally characterized by the presence of seven transmembrane domains and their intracellular association with G-proteins (for a review, see reference 48). The binding of an extracellular ligand to the receptor initiates intracellular signaling by stimulating the associated heterotrimeric G-proteins to exchange GDP for GTP, causing them to dissociate into the GTP-bound Gα subunit and a Gβ/Gγ dimer, each of which functions in the activation or inactivation of specific pathways (for a review, see reference 48). There are three *A. fumigatus* Gα proteins (GpaA, GpaB, and GpaC) (49). There are several phenotypes associated with the *gpaB* deletion mutant, such as a significantly decreased germination rate, conidiation, mRNA expression of critical asexual development regulators, a reduction in conidial tolerance against H_2_O_2_, but not to paraquat, and an enhanced susceptibility against membrane-targeting azole antifungal drugs and reduced production of gliotoxin (49). Here, we demonstrate that GpaB also affects the dual biofilm interaction Af×Pa by decreasing biofilm formation, specifically in the presence of *P. aeruginosa*.

Interestingly, *P. aeruginosa* has increased growth and adhesion to the surface of the germlings in the presence of the Δ*gpaB* mutant, suggesting that GpaB is essential for maintaining a growth equilibrium between the two partners during dual biofilm interaction. The same behavior is not observed during ΔgpaB×Pa interaction, suggesting GpaB is controlling more functions involved in the interaction than pyripyropene production. GpaB affects the MAPK MpkA phosphorylation and PKA activity. It has been previously reported that *A. fumigatus* MpkA is important for pyripyropene production (26), but it remains to be investigated if PKA is involved in the modulation of pyripyropene production. Even more relevant is the fact that the production and secretion of fumiquinazoline and pyripyropene are not impacted by the lack of *gpaB* during biofilm formation but only during dual biofilm interaction in the presence of *P. aeruginosa*. Fumiquinazolines are tryptophan-derived peptidyl alkaloids, and besides their secretion, they also accumulate in *A. fumigatus* conidia (50). It is known that *Penicillium coryphilum* fumiquinazoline F has inhibitory activity against *Staphylococcus aureus* and *Micrococcus luteus* (51). *Acremonium sp.* fumiquinazolines H and I also have inhibitory activity against *Candida albicans* (52). It remains to be investigated how fumiquinazole F/G is affecting the Af×Pa biofilm dual formation.

We investigated the role played by pyripyropenes on *A. fumigatus* versus *P. aeruginosa* dual biofilm formation. Pyripyropenes are the most potent known inhibitors of mammalian ACATs or sterol O-acyltransferases (SOAT; [19–21]). Previous studies have shown that ACATs are endoplasmic reticulum (ER)-localized multi-transmembrane proteins that are evolutionarily conserved from yeast to humans (45). ACATs catalyze the reaction between long-chain fatty acyl-CoA and intracellular cholesterol or ergosterol forming cholesteryl or ergosteryl esters that will be stored in lipid droplets into the cell or transported in secreted lipoprotein particles when the cells need available cholesterol or ergosterol (53). Although *S. cerevisiae* SOATs have already been characterized, to our knowledge, there is no demonstration that pyripyropene can inhibit fungal SOATs (43, 54). Pyripyropenes have a narrow interspecies distribution pattern, what highlights the importance of this SM in the Af×Pa biofilm interaction. Curiously, other fungal BGC-producing ACAT inhibitors have evolved, such as beauveriolides produced by *Beauveria* spp. and *Cordyceps militaris*, 7-chlorofolipastatin by *Aspergillus unguis*, and helvamide by *Aspergillus nidulans* (55–59), suggesting that the strategy of producing diverse ACAT inhibitors different from pyripyropenes has evolved independently a few times. It has been previously determined that pyripyropenes showed no *in vitro* antimicrobial activity at a concentration of 1 mg/mL (1.77 mM) against *P. aeruginosa,* as also shown here, and several other bacteria and fungi, such as *Bacillus subtilis, Mycobacterium smegmatis, P. aeruginosa, Escherichia coli, Micrococcus luteus, Staphylococcus aureus, Candida albicans, Saccharomyces cerevisiae, Pyricularia oryzae, Mucor racemosus*, and *A. niger* (20).

We deleted the *A. fumigatus pyr2* gene that encodes a non-reducing polyketide synthase and pyripyropene production, which was completely abolished *in vitro* and during the dual biofilm Af×Pa interaction. We observed that the lack of *pyr2* during the dual biofilm interaction affected the production of several lipids, including ergosterol, and influenced the sphingolipid metabolism, suggesting a possible role for pyripyropene as a fungal modulator of ergosterol and lipid metabolism. We also constructed null mutants for ACAT homologs, *acaA* and *acaB*, and both mutants have reduced growth during Af×Pa dual biofilm formation, but not a single biofilm formation when compared to the wild-type strain. The reduction in *A. fumigatus* and *P. aeruginosa* mixed biofilm formation with the Δ*acaA* and Δ*acaB* mutants is less pronounced compared to the phenotype observed with Δ*gpaB*, what suggests a functional redundancy between these two genes, and this remains to be investigated. Although not proven here, it is tempting to speculate that pyripyropenes could modulate AcaA and AcaB activities. We have also not observed a distinct *P. aeruginosa* transcriptional signature when the bacteria are exposed to pyripyropene, which suggests that pyripyropene does not directly affect *P. aeruginosa*. We could not provide a direct explanation of how pyripyropene is affecting the Af×Pa dual biofilm interaction. However, since pyripyropene is affecting ergosterol and lipid metabolism, it is possible the cell membrane composition is different in the *pyr2* and *acaA* and *acaB* null mutants, and this could contribute to the incorrect assembly of essential cell membrane proteins as well as the fusion and deposition of vesicles containing precursors required for cell wall growth that are transported to the hyphal tip through a network of microtubules and the actin cytoskeleton (60, 61). Changes in cell membrane composition and organization also affect protein secretion, endocytosis, and the proper deposition of components of the cell wall and plasma membrane (for a review, see reference 62). This could also influence the anchorage of the cell wall to the plasma membrane, affecting either cell wall morphology or its adherence to the plasma membrane (for reviews, see references 63–65). All these changes do not affect the growth of *P. aeruginosa* but could contribute to less efficient *A. fumigatus* growth during Af×Pa dual biofilm formation.

Notably, the Δ*pyr2* mutants have attenuated virulence in a murine chemotherapeutic model of IPA, suggesting pyripyropene biosynthesis is impacting not only the Af×Pa biofilm formation but also the establishment and further colonization of murine lungs. The Δ*pyr2* mutants also have reduced survival in the presence of BMDMs and inflammatory cytokines when compared to the wild-type strain. The reduced survival and cytokine production suggest that lack of pyripyropene production is affecting the conidial cell wall structure and organization. Considering Δ*pyr2* mutants have the same virulence as the wild-type strain in a murine immunocompetent model, these results with macrophages suggest pyripyropene is important for the initial steps of the infection as a mechanism of evasion and modulation of the host immunity. In contrast, maybe the leading cause for the attenuation of Δ*pyr2* mutants in the murine chemotherapeutic model is related to a possible reduction in *A. fumigatus* fitness caused by the loss of the ability to produce pyripyropene. However, since pyripyropene is an inhibitor of cholesterol esterification, it is also possible that this SM is affecting cholesterol metabolism in animal cells. There is a correlation between the overexpression of human ACAT1 and increased cholesteryl ester accumulation in LDs in a variety of cancer types (66). There is also a preference for the formation of CEs for storage in cancer cells, and it has also been shown that breast cancer cell lines (MDA-MB-436 and MDA-MB-231) contain a greater number of cytoplasmic LDs than luminal MCF-7 breast cancer cell line (Michigan Cancer Foundation) (67). The Δ*pyr2* mutant triggers significantly reduced levels of LDs when compared with the wild-type, providing initial evidence that *A. fumigatus* pyripyropene secretion is affecting cholesterol metabolism. The homeostasis of cholesterol in the plasma membranes of animal cells is a process controlled through its interactions with phospholipids and transmembrane domains of proteins (68). The traffic of cholesterol between the plasma membrane and the endoplasmic reticulum is essential for cholesterol homeostasis. For example, the oxysterol 25-hydroxycholesterol activates ACAT in the endoplasmic reticulum, stimulating the internalization of accessible cholesterol from the plasma membrane (69).

Interestingly, this immunomodulatory oxysterol pathway has been demonstrated as important for containing bacterial and viral pathogens and pore-forming toxins (69–71). To our knowledge, there are no studies in human fungal pathogens, including *A. fumigatus*, about the effects of cholesterol removal of the plasma membranes and further esterification upon fungal infection. However, the increased susceptibility of hyperlipoproteinemic, apolipoprotein E (ApoE)-deficient (ApoE−//−) mice to a systemic *Candida albicans* infection has been demonstrated (72). Mortality due to candidemia was significantly higher in ApoE−//−/ mice than in ApoE+//+/ mice, suggesting that lipoproteins play a significant role in host defense against candidiasis (72). It remains to be determined if *A. fumigatus* pyripyropene is inhibiting animal ACATs and not allowing cholesterol removal from the plasma membranes. This could be a novel mechanism of *A. fumigatus* evasion and host immunity modulation.

## MATERIALS AND METHODS

### *P. aeruginosa* and *A. fumigatus* strains and growth conditions

All *A. fumigatus* strains used in this work were described in Table S9 (https://doi.org/10.6084/m9.figshare.28304309). Strains were grown at 37°C. Conidia of *A. fumigatus* were grown from frozen stocks on MM (1% [wt/vol] glucose, nitrate salts, trace elements, pH 6.5). For solid minimal medium, 2% agar was added. Trace elements and nitrate salt compositions were as described previously (73). Conidia suspensions were obtained by harvesting grown mycelia on minimal medium plates as described by Ries and colleagues (74). For phenotypic characterization, plates were inoculated with 10^4^ spores per strain and left to grow for 120 h at 37°C. Radial growth experiments were expressed as ratios, dividing the colony radial diameter of growth in the stress condition by the colony radial diameter in the control (no stress) condition. *P. aeruginosa* was grown from frozen stocks (Luria-Bertani [LB] medium plus 20% glycerol) in solid LB for 24 h at 37°C. A single colony was transferred to 30 mL of LB and cultured overnight at 37°C at 200 rpm. The culture was centrifuged at 4,000 × *g* for 5 min, and the pellet was washed with 10 mL of phosphate-buffered saline (PBS). After centrifugation, the pellet was resuspended in LB and the inoculum was adjusted, using a spectrophotometer, to an optical density at 600 nm (OD_600_) of 0.07 to 0.075. This inoculum was grown in 30 mL of LB at 37°C at 200 rpm for 5 h, and the centrifugation and PBS washing processes were repeated. The final pellet was resuspended in RPMI-HEPES, and the inoculum was adjusted to an OD of 0.07 to 0.075 (approximately 5 × 10^5^ to 8 × 10^6^ CFU/mL).

### Generation of *A. fumigatus* mutants

All gene replacement cassettes were constructed by “*in vivo*” recombination in *S. cerevisiae*, as previously described by reference 75. For the construction of A. *fumigatus* Δ*pyr 2.1*, Δ*pyr 2.2*, Δ*acaA*, and Δ*acaB*, approximately 1.0 kb from each 5′ untranslated region (5′-UTR) and 3′-UTR flanking regions of the targeted open reading frame (ORF) regions were selected for primer design. The primers gene_pRS426_5UTR_fw and gene_pRS426_3UTR_rv contained a short homologous sequence to the multiple cloning site (MCS) of the plasmid pRS426. The 5- and 3-UTR fragments were PCR-amplified from the genomic DNA of *A. fumigatus* CEA17, *pyrG*^+^ strain. The *pyrG* gene placed within the cassette as a prototrophic marker was amplified from the pCDA21 plasmid (76) using the primers described in Table S10 (https://doi.org/10.6084/m9.figshare.28304309). The cassette was PCR-amplified from these plasmids utilizing TaKaRa Ex Taq DNA Polymerase (Clontech Takara Bio) and used for *A. fumigatus* transformation. Southern blot analysis was performed to confirm the deletions (Fig. S2 at https://doi.org/10.6084/m9.figshare.28304309).

### Checkerboard assay

Checkerboard assays were performed to assess the interaction (synergistic, additive, or antagonistic) between the selected compounds and pyripyropene. Briefly, a stock solution of 2.5 × 10^4^ conidia/mL and 5 µg/mL of pyripyropene, 8 µg/mL of voriconazole, and 6 0 µg/mL of cerulenin were prepared in MM supplemented with 1% of glucose or 0.01% of ergosterol with 10% Alamar blue. In 96-well microtiter plates, voriconazole or cerulenin was diluted sequentially along the ordinate, while the pyripyropene was diluted along the abscissa to obtain a final volume of 100 µL. The plates were incubated for 48 h at 37°C, and the metabolic activity was determined by reading in the spectrophotometer as previously described. Results are expressed as means SD from two independent experiments. To determine the type of drug interaction, the SynergyFinder software (77) was used with the following parameters: detect outliers: yes; curve fitting: LL4; method: Bliss; correction: on. The summary synergy score represents the average excess response due to drug interaction, in which a value less than −10 suggests an antagonistic interaction between two drugs; values from −10 to 10 indicate an additive interaction, and values more significant than 10 suggest a synergistic interaction.

### RNA extraction, complementary DNA (cDNA) synthesis, and RT-qPCR

All experiments were carried out in biological triplicate, and conidia (10^7^) were inoculated in the liquid culture medium (MM [with or without glucose treatment]). For total RNA isolation, mycelia were ground in liquid nitrogen, and total RNA was extracted using TRIzol (Invitrogen), treated with RQ1 RNase-free DNase I (Promega), and purified using the RNAeasy kit (Qiagen) according to the manufacturer’s instructions. RNA was quantified using a NanoDrop. The RNA was reverse transcribed to cDNA for RT-qPCR using the ImProm-II reverse transcription system (Promega) according to the manufacturer’s instructions. The synthesized cDNA was used for real-time analysis using the SYBR Green PCR master mix kit (Applied Biosystems) in the ABI 7500 Fast real-time PCR system (Applied Biosystems, Foster City, CA, USA). SYBR primer sequences are listed in Table S10 at https://doi.org/10.6084/m9.figshare.28304309). The actin gene was used as a normalizer.

### Phylogenetic analysis

We used the amino acid sequences of genes in the *A. fumigatus* pyripyropene BGC to query proteomes of Ascomycota species in the (78) data set using diamond (v.2.1.9.163 [79]) blast (v.2.13.0), as well as to query the nr and RefSeq databases using cblaster (v.1.0) to get an overview of potential BGC presence (80). The results were filtered, requiring the presence of *pyr2*, a minimum of five of the genes in proximity (--min_identity 30 --min_coverage 50), in an assembled genome. For extracting exact gene positions, hit quality metrics, and directions/orientation, we downloaded the assemblies and re-ran a Diamond BLAST and TBLASTN searches locally. The genomes putatively containing the BGC were assessed with BUSCO (v.4.5.6 [81]) with the lineage set fungi_obd10 (2014-01-08). We used BUSCOs (https://busco.ezlab.org/) to search for ortholog nucleotides present in all genome sequences (v.2.4.0 [82]), aligning them with mafft (v.7.505 [83]) applying default settings. The phylogeny of the species was inferred based on these nucleotide alignments using iqtree2 (v.2.2.0.3 [84–86]) and 1,000 ultrafast bootstrap replicates. The individual BGC gene trees were inferred from amino acid sequence alignments produced in previous diamond blast and tblastn searches and were aligned using muscle5 (87), producing a stratified ensemble alignment (-align -stratified) and selecting the alignment with the highest column confidence (-maxcc). All data analyses and visualizations were done in R version 4.4.0, mainly using the packages tidyverse, ggtree, and gggenes. For visualizing supplementary phylogenies, FigTree v.1.4.4 was used, rerooting most phylogenies with the clade of *Hirsutella*.

### Dual biofilm formation between *P. aeruginosa* and *A. fumigatus*

To measure the interaction between *P. aeruginosa* and *A. fumigatus* and to determine the SM produced by them in single or cocultures, 1 × 10^5^ CFU/mL of *P. aeruginosa* were inoculated with or without 1 × 10^6^ conidia/mL of *A. fumigatus* in 20 mL of RPMI-HEPES medium into polystyrene Petri dishes at 37°C. After 5 days, the supernatant was collected, the plate was washed with 10 mL of ultrapure water to collect the cells, and both were transferred to a 50 mL tube. This mixture was centrifuged at 4,000 × *g* for 15 min at 4°C to obtain the pellet, which was used for the dual quantification of species-specific biofilm growth by qPCR, with primer for *ecfX* and 18S, as described by Bastos and colleagues (18), and the supernatant (20 mL), which was filtered through a 0.22 µm filter, was frozen and lyophilized for SM extraction. Biofilm production was also measured by the CV method in 96-well plates, under hypoxic (1% O_2_, 5% CO_2_) or normoxic (approximately 20% O_2_ and 0.04% CO_2_) conditions, at 37°C. *A. fumigatus* strains (1 × 10^6^ conidia/mL) were inoculated with or without *P. aeruginosa* (1 × 10^5^ CFU/mL) in 200 µL of RPMI-HEPES for 48 h. The biofilm was dried at 37°C for 30 min and then stained with 5 mL of 0.05% (wt/vol) CV for 10 min. The plates were washed with 50 mL of PBS, and the CV was solubilized with 3 mL of 95% ethanol. Samples of 100 µL were transferred to a new 96-well plate, and the absorbance at 595 nm was determined, as a measure for biofilm formation.

### RNAseq for *P. aeruginosa* exposed to pyripyropene

The *P. aeruginosa* PA14 strain was cultured on *Pseudomonas* isolation agar plates overnight at 37°C. Individual colonies were selected and inoculated into RPMI-HEPES medium, followed by overnight incubation under shaking conditions at 37°C. After overnight culture, resuspended pellets were diluted to an OD_600_ of 0.05 in fresh RPMI-HEPES medium (6 mL). The cultures were incubated statically at 37°C until mid-exponential phase. Experimental samples were treated with pyripyropene A at a final concentration of 10 µg/mL, while control samples received an equal volume of dimethyl sulfoxide. Following treatment, the cultures were incubated for an additional 3 h at 37°C. The cultures were removed and immediately added to an equal volume of RNAlater solution (Invitrogen) to preserve RNA integrity. The cells were then harvested by centrifugation at 4,000 × *g* for 10 min at room temperature (RT). The resulting pellet was resuspended in 10 mM Tris buffer + 50 mg/mL lysozyme. RNA was extracted from the harvested cells using the Zymo Quick RNA Fungal/Bacterial Miniprep Kit (Zymo Research) according to the manufacturer’s instructions. The quality and quantity of RNA were assessed using a NanoDrop spectrophotometer and agarose gel electrophoresis. RNA samples were sent to SeqCenter for sequencing using the Illumina Stranded RNA library preparation with RiboZero Plus rRNA depletion kit. Default parameters were used for all software. Trimmed reads were then mapped to *P. aeruginosa* PA14 reference genome using Bowtie2 v.2.4.2 with default parameters for end-to-end alignment. Read summarization was performed using featureCounts. DESeq2 was employed to analyze differentially expressed genes. Annotations of differentially expressed genes were obtained from the reference annotation of the *Pseudomonas* genome available at the Pseudomonas Genome Database website. Genes were considered significantly altered when their adjusted *P*-value was <0.05.

### RNA purification and preparation for RNA-seq

Total RNAs of cultures of early dual biofilm interactions of 24 and 48 h were extracted by the TRIzol method, treated with RQ1 RNase-free DNase I (Promega), and purified using the RNAeasy kit (Qiagen) according to the manufacturer’s instructions. The total RNA was quantified using a NanoDrop, and its integrity was analyzed using an Agilent 2100 Bioanalyzer. All RNA had a minimum RNA integrity number value of 8.0. For RNA sequencing, the cDNA libraries were constructed using the TruSeq Total RNA with Ribo Zero (Illumina, San Diego, CA, USA). From 0.1 to 1 µg of total RNA, the ribosomal RNA was depleted, and the remaining RNA was purified, fragmented, and prepared for cDNA synthesis, according to manufacturer recommendations. The libraries were validated following the Library qPCR Quantification Guide (Illumina). Following, the RNA-seq was carried out by paired-end sequencing on the Illumina NextSeq 500 Sequencing System using NextSeq High Output (2 × 150) kit, according to manufacturer recommendations.

### Macrophage culture

BALB/c BMDMs were obtained as previously described (88). Briefly, bone marrow cells were cultured for 7–9 days in RPMI 20/30, which consists of RPMI-1640 medium (Gibco, Thermo Fisher Scientific Inc.), supplemented with 20% (vol/vol) fetal bovine serum (FBS) and 30% (vol/vol) L-cell conditioned media as a source of macrophage colony-stimulating factor on non-treated Petri dishes (Optilux-Costar, Corning Inc., Corning, NY). Twenty-four hours before experiments, BMDM monolayers were detached using cold PBS (Hyclone, GE Healthcare Inc., South Logan, UT) and cultured, as specified, in RPMI-1640 (Gibco, Thermo Fisher Scientific Inc.) supplemented with 10% (vol/vol) FBS, 10 U/mL penicillin, and 10 mg/mL streptomycin, (2 mM) L-glutamine, (25 mM) HEPES, pH 7.2 (Gibco, Thermo Fisher Scientific Inc.), at 37°C in 5% (vol/vol) CO_2_ for the indicated periods.

### Macrophage infection, cytokine, and LDH determination

BMDMs were cultured as described before and were seeded at a density of 1 × 10^6^ cells/mL in 24-well plates (Greiner Bio-One, Kremsmünster, Austria). The cells were challenged with the conidia of different strains at a multiplicity of infection of 1:10 and incubated at 37°C with 5% (vol/vol) CO_2_ for 24 h. BMDMs were also stimulated with different concentrations of AspA protein (denatured or not) by boiling for 10 min at 100°C. Lipopolysaccharide (LPS; standard LPS, *E. coli* 0111: B4; Sigma-Aldrich, 500 ng/mL) plus nigericin (tlrl-nig, InvivoGen, 5 µM/mL) and medium alone were used, respectively, as the positive and negative controls. Cell culture supernatants were collected and stored at −80°C until they were assayed for TNF-α, IL-1, and LDH release using Mouse DuoSet enzyme-linked immunosorbent assay (ELISA) kits (R&D Systems, Minneapolis, MN, USA) and CyQUANT LDH Cytotoxicity Assay (Invitrogen), according to the manufacturer’s instructions. For cytokine determination, plates were analyzed by using a microplate reader (Synergy HTX Multi-Mode, BioTek) measuring absorbance at 450 nm. Cytokine concentrations were interpolated from a standard curve, and statistical significance was determined using an analysis of variance (ANOVA) (GraphPad Prism 8.0, La Jolla, CA). The level of LDH was determined by measuring absorbance at 490 and 680 nm using a microplate reader (Synergy HTX Multi-Mode, BioTek). All assays were performed in triplicate in three independent experiments.

### *Aspergillus* growth and fluorescein isothiocyanate (FITC) label

*A. fumigatus* strains were cultivated on MM agar plates at 37°C for 3 days. Conidia were harvested in sterile water with 0.05% (vol/vol) Tween 20. The resulting suspension was filtered through two layers of gauze (Miracloth, Calbiochem). FITC labeling of conidia was performed with 0.1 mg/mL FITC (Sigma) in 0.1 M Na_2_CO_3_ at 37°C for 30 min. Labeled conidia were washed three times with PBS, 0.1% (vol/vol) Tween 20. The conidia concentration was determined using a hemocytometer.

### Phagocytosis and adhesion assays

BMDMs were cultivated in Dulbecco’s modified Eagle medium (DMEM) supplemented with 10% (vol/vol) heat-inactivated fetal calf serum, 2 mM glutamine, and penicillin-streptomycin. For infection experiments, macrophages were seeded on glass cover slips in 24-well plates at a density of 5 × 10^5^ cells per well and allowed to grow adherently overnight. Following washing with pre-warmed medium, FITC-labeled conidia were added at a multiplicity of infection of 10. The infection experiment was synchronized for 30 min at 4°C. Unbound conidia were removed by washing with pre-warmed medium, and phagocytosis was initiated by shifting the co-incubation to 37°C in a humidified CO_2_ incubator. After 1 h, the phagocytosis was stopped by washing with ice-cold PBS. Labeling of extracellular conidia was performed by incubation with PBS and 0.25 mg/mL calcofluor white (Sigma) for 30 min at 4°C. The cells were washed twice with PBS and fixed with 3.7% (vol/vol) formaldehyde/PBS for 15 min followed by two washes with PBS. Microscopic photographs were taken on a Zeiss microscope. For statistical reproducibility, two biological replicates and, in each case, two technical replicates were made and analyzed for each strain. The phagocytic index was enumerated by counting 100 macrophages per cover slip from duplicate wells. The phagocytic index was calculated by the average number of conidia that had been phagocytosed for each macrophage.

### Conidial killing assay

BMDMs were seeded at a density of 10^6^ cells/mL in 24-well plates (Corning Costar) and were challenged with conidia at a multiplicity of infection of 1:10 and incubated at 37°C with 5% (vol/vol) CO_2_ for 24 h. After the incubation media were removed, the cells were washed with ice-cold PBS, and finally, 2 mL of sterile water was added to the wells. A P1000 tip was then used to scrape away the cell monolayer, and the cell suspension was collected. This suspension was then diluted 1:1,000, and 100 µL was plated on Sabouraud agar before the plates were incubated at 37°C overnight, and the colonies were counted. Fifty microliters of the inoculum adjusted to 10^3^/mL was also plated on Sabouraud (SAB) agar to correct CFU counts. The CFU/mL for each sample was calculated and compared to the A1163 wild-type strain.

### Animal survival curves

Inbred female mice (BALB/c; body weight, 20 g–22 g; age of 6 to 8 weeks) were housed in vented cages containing five animals. Cages are well-ventilated, softly lit, and subjected to a 12:12 light:dark cycle. The relative humidity was kept at 40 to 60%. Mouse room and cages were kept at a temperature range of 22°C. Mice were immunosuppressed with cyclophosphamide (150 mg/kg of body weight), which was administered intraperitoneally on days −4, −1, and 2 prior to and post-infection (infection day is “day 0”). Hydrocortisone acetate (200 mg/kg body weight) was injected subcutaneously on day −3. Mice (10 mice per group, two repetitions) were anesthetized by halothane inhalation and infected by intranasal instillation of 20 µL containing 1.0 × 10^5^ conidia of *A. fumigatus* wild-type or mutant strains (the viability of the administered inoculum was determined by incubating different serial dilutions of the conidia used in both repetitions on MM, at 37°C). As a negative control, a group of 10 mice received PBS only. Animals were sacrificed 15 days post-infection or if moribund. To investigate animal survival, mice (10 [immunosuppressed model] or 5 [immunocompetent model] mice per group, two repetitions) are immunosuppressed as described previously or not, and mice were intranasally inoculated with 1 × 10^6^ conidia/20 µL of suspension for the chemotherapeutic murine model and with 5 × 10^8^ conidia/20 µL for the immunocompetent murine model.

### Cytokine and chemokine quantification

For lung homogenates, the lungs of all experimental groups were homogenized in PBS supplemented with Complete Mini protease inhibitor tablets (Roche), clarified by centrifugation, and stored at −80°C. A panel of cytokines and chemokines was quantified by ELISA (R&D Systems) according to the manufacturer’s instructions.

### Macrophage lipid droplet formation

Bone marrow-derived macrophages (BMDMs) were cultured in DMEM as previously described (88) and seeded at a density of 1 × 10^6^ cells/mL in glass-bottom cell culture dish (CellView). BMDMs were or not challenged with 1 × 10^5^ conidia of wild type and Δ*pyr2* and incubated at 37°C with 5% (vol/vol) CO_2_ for 24 h. After incubation, media was removed and 2 µM BODIPY 493/503 (Invitrogen) staining solution in PBS was added for 15 min at 37°C. Cell culture dishes were visualized on a Carl Zeiss Observer Z1 fluorescence microscope using the excitation wavelength of 450 to 490 nm and emission wavelength of 500 to 550 nm. Differential interference contrast images and fluorescent images were captured with an AxioCam camera (Carl Zeiss) and processed using AxioVision software (version 4.8). The percentage of macrophages with lipid droplets was determined. The results were expressed as (%) lipid droplets per number of cells in each microscopy field. Two independent experiments were performed, and 28 microscopy fields with approximately 700 BMDMs in total were counted.

### PKA activity assay

Single or cocultures of *P. aeruginosa* and *A. fumigatus* were grown in RPMI-HEPES medium into polystyrene Petri dishes (as described above) for 96 h at 37°C. Cell pellets were then snap-frozen in liquid nitrogen, and the total protein content was extracted by grinding the pellets using mortar and pestle in liquid nitrogen and resuspended in lysis buffer present in the kit (Tris-based, pH 8 buffer containing 1% NP-40) supplemented with an EDTA-free Protease Inhibitor Cocktail tablet (Roche), phenylmethanesulfonyl fluoride (1 mM final concentration), and 50 µL activated sodium orthovanadate (10 mM final concentration). Extracts were centrifuged at 13,000 × *g* for 20 min at 4°C. The supernatants were collected, and the total protein abundance was determined using the 2-D Quant Kit (GE Healthcare Life Sciences). The PKA Colorimetric Activity Kit (EIAPKA, ThermoFisher Scientific) was performed according to the manufacturer’s instructions. The endpoint reaction was detected using a Synergy-HT microplate reader (Bio-Tek) at 450 nm. PKA activity was determined relative to the total protein content of the samples.

### Western blot analysis

Single or cocultures of *P. aeruginosa* and *A. fumigatus* were grown under the same conditions as described for the PKA activity assay, and the total cellular protein extractions were carried out. The supernatants were collected, and the total protein abundance was determined using Bradford reagent (Bio-Rad), according to manufacturer’s instructions. Fifteen micrograms of protein from each sample was resolved in a 12% (wt/vol) SDS-PAGE and transferred to polyvinylidene difluoride membrane (Merck Millipore). The phosphorylated fractions of the MAPK (P-MpkA) and the total MpkA were examined using anti-phospho p44/42 MAPK and p44/42 MAPK antibodies (Cell Signaling Technologies), respectively, following the manufacturer’s instructions using a 1:5,000 dilution. The histone was identified using anti-histone H3 antibody (Abcam, ab1791). The primary antibody was detected using a horseradish peroxidase (HRP)-conjugated secondary antibody raised in rabbit (Sigma). Chemiluminescent detection was achieved using an ECL Prime Western Blot detection kit (GE HealthCare). To detect these signals on blotted membranes, the ECL Prime Western Blotting Detection System (GE Healthcare, Little Chalfont, UK) and LAS1000 (FUJIFILM, Tokyo, Japan) were used. The quantification of the signal intensity ratio of P-MpkA/H3 or MpkA/H3 was performed using ImageJ.

### Biofilm imaging

*P. aeruginosa* was grown from frozen stocks ([LB] medium plus 20% glycerol) in 1 mL LB for 24 h at 30°C. *A. fumigatus* 1 × 103 conidia were grown in eight-well glass-bottom slides (Ibidi) with 500 µL RPMI-HEPES medium at 30°C for 7 h. The culture medium of *P. aeruginosa* was diluted with sterile water to an OD_600_ of 0.01, and 10 µL was added to the *A. fumigatus*-precultured chamber. The eight-well chamber was incubated for 24 h at 30°C. The medium was removed by pipetting. The biofilm samples on the glass surface were fixed with 500 µL PBS containing 1% glutaraldehyde and 4% paraformaldehyde for 30 min at room temperature. The fixed solution was removed by pipetting. The biofilm samples on the glass surface were washed with PBS. *P. aeruginosa* cells were stained with 1,000 times diluted SYBR Green (TAKARA, Japan) in PBS. *A. fumigatus* cells were stained with ConA-Alexa 594 (Invitrogen), stock solution at 5 mg/mL was diluted 10,000 times in PBS. The staining solution was replaced with 200 µL iCBiofilm clearing reagent-H1 (Tokyo Chemical Industry, Japan) to make the biofilm transparent (89). The biofilms were observed using fluorescent microscopy THUNDER DMi8 (Leica Microsystems) with a 20× objective lens (NA 0.8) in SVCC mode. Z-stacks were taken at 1 µm intervals. Images were collected and analyzed by the LAS X system (Leica) and ImageJ software. Biofilm volumes were measured from the 3D images using Volocity software. The laser intensity and exposure time were set constant to avoid saturation of the signal when taking green or red images with the THUNDER fluorescence microscope, respectively. The 3D images were opened in Volocity software, and the range of green or red signal intensity was set to a constant value while checking the images to ensure that the extracted signals were appropriate for the 3D signal volume.

### SM extraction and LC-HRMS/MS analysis

SMs were extracted from 50 mg freeze-dried sample of the entire supernatant of each sample by resuspension in 1 mL of HPLC-grade methanol (MeOH), followed by 1 h of sonication in an ultrasonic bath. For sample preparation, 500 µL of each obtained extract was filtered (0.22 µm filter), transferred to vials, and diluted with HPLC-grade MeOH to a total volume of 1 mL.

LC-HRMS/MS positive-mode analysis was performed in a Thermo Scientific QExactive hybrid Quadrupole-Orbitrap mass spectrometer coupled to a Dionex UltiMate 3000 RSLCnano UHPLC system. For the stationary phase, a Thermo Scientific column, Accucore C_18_ 2.6 µm (2.1 mm by 100 mm) was used. The mobile phase was 0.1% formic acid (A) and acetonitrile plus 0.1% formic acid (B). Eluent profiles (A/B percentages) were 95/5 for the first 5 min of separation, up to 60/40 at 10 min, 55/45 was reached at 12 min and 2/98 at 18 min, being maintained for 2 min. The initial 95/5 was reached at 22 min and kept for an extra 2 min for column conditioning for the next injection. Total run time was 24 min for each run, and flow rate was 250 µL/min. The injection volume was 5 µL. The chromatographic column was maintained at 40°C during analysis. MS spectra were acquired with *m/z* ranges from 100 to 1,500, with 70,000 for mass resolution at 200 Da. Ionization parameters were sheath gas flow rate of 45 L/h, auxiliary gas flow rate of 10 L/h, sweep gas flow rate of 2 L/h, spray voltage of 3.5 kV, capillary temperature of 250°C, S-lens RF level of 50, and auxiliary gas heater temperature of 400°C. MS/MS spectra were acquired in data-dependent acquisition mode. Normalized collision energy was applied stepwise (20, 30, and 40 V), and the five most intense precursors per cycle were measured with 17,500 resolution at 200 Da.

### LC-HRMS/MS data processing, feature-based molecular networking (FBMN), and *in silico* structure prediction

Raw LC-HRMS/MS data were converted into mzXML format files using MSConvert (90), with 32-bit binary encoding precision, zlib compression, and peak peaking. Feature detection was performed in MZmine (v.3.4.16) (91). For MS spectra mass detection, an intensity threshold of 1E4 was used, and for MS/MS, an intensity threshold of 1E2 was used. For chromatogram building (92), scans were filtered based on retention time, maintaining scans acquired from 2 to 20 min. A 5 ppm mass accuracy, a minimum intensity for four consecutive scans of 1E4, and a minimum peak intensity of 3E4 were set. Extracted ion chromatograms (XICs) were deconvolved using the baseline cut-off algorithm at an intensity of 1E5, minimum peak height of 3E4, and a peak duration range from 0.02 to 3 min. After chromatographic deconvolution, XICs were matched to MS/MS spectra within *m/z* 0.01 and 0.1 min retention time windows. Isotope peaks were grouped with 3 ppm mass tolerance, 0.04 min retention time tolerance, and a maximum charge of 2. Detected peaks in different samples were aligned with a 5 ppm tolerance, 75% wt for *m/z* determinations, and 25% for retention time. MS features without MS/MS features assigned were filtered out of the resulting matrix as well as features that did not occur in at least three samples. Finally, the feature table was exported as a .csv file, and corresponding MS/MS spectra were exported as .mgf files. Features observed in blank samples were filtered.

A molecular network was created with the FBMN workflow (93) on GNPS (https://gnps.ucsd.edu) (30). The data were filtered by removing all MS/MS fragment ions within 17 Da of the precursor *m/z*. MS/MS spectra were window filtered by choosing only the top 6 fragment ions in the ±50 Da window throughout the spectrum. The precursor ion mass tolerance was set to 0.02 Da, and the MS/MS fragment ion tolerance was set to 0.02 Da. A molecular network was then created in which edges were filtered to have a cosine score above 0.7 and more than five matched peaks. Furthermore, edges between two nodes were kept in the network if and only if each of the nodes appeared in each other’s respective top 10 most similar nodes. Finally, the maximum size of a molecular family was set to 100, and the lowest-scoring edges were removed from molecular families until the molecular family size was below this threshold. The spectra in the network were then searched against GNPS spectral libraries (30, 94). The library spectra were filtered in the same manner as the input data. All matches kept between network spectra and library spectra were required to have a score above 0.7 and at least five matched peaks. The molecular networks were visualized using Cytoscape software (95). Resulting networks were displayed and analyzed with Cytoscape (v.3.8.2).

The exported .mgf file was analyzed with SIRIUS (v.5.7.0) (32) using the default Orbitrap parameters to predict feature molecular formulae and fragmentation trees. Candidate formulas were then reranked with ZODIAC (96). CSI:FingerID (31) was applied for molecular fingerprint prediction based on MS/MS spectra, being then queried with the Bio Database.

For SM dereplication, metabolites were annotated based on library matching with the GNPS MS/MS database via the FBMN workflow and through *in silico* fingerprint prediction and comparison with structure databases in the SIRIUS environment.

### Pyripyropene A detection in *in vitro* infection of BMDMs

SMs were extracted from freeze-dried samples of 24 h infected (multiplicity of infection [MOI] 1:10) BMDMs (1 × 10^7^ cells) by resuspension in 500 µL of HPLC-grade methanol (MeOH), followed by 1 h of sonication in an ultrasonic bath. For sample preparation, obtained extracts were filtered (0.22 µm filter) and transferred to vial inserts. A pyripyropene A chemical standard was acquired, and a solution with a final concentration of 0.1 ppm was prepared in HPLC-grade MeOH. LC-MS analyses were performed in an UPLC ACQUITY (Waters) coupled to a Xevo Triple Quadrupole–TQD (Waters) with an electrospray ionization source. Chromatographic separation was achieved with an ACQUITY UPLC BEH C18 (Waters) (2.1 mm × 50 mm, 1.7 µm) column maintained at 40°C with a 400 µL/min flow rate. Isocratic elution was performed with a 70/30% acetonitrile/water mixture over 4 min. Mass spectra were acquired in positive ionization mode in a selected reaction monitoring experiment set to monitor the *m/z* 584.2 > *m/z* 148.0 transition. Identification of pyripyropene A was confirmed by matching retention time and mass spectra between data acquired from the pyripyropene A chemical standard and biological samples.

### Analysis in scan mode by GC-MS

For GC-MS analysis, 50 µL of each sample was mixed in a vortex with 150 µL of ice-cold methanol in a 3:1 ratio (methanol:sample) ensuring total deproteinization, and then centrifuged for 10 min at 10,000 × *g* at room temperature. One hundred microliters of the supernatant was transferred to GC-MS analysis vials containing glass inserts, and then evaporated in SpeedVac at 30°C. For methoximation of the samples, 15 µL of O-methoxyamine hydrochloride (15 mg/mL in pyridine) was added to each analysis vial and then taken to three later cycles of vortexing for 2 min and ultrasound in water for 40 s. The vials were covered and incubated in a dark environment at room temperature for 16 h. After incubation, 15 µL of *N*,*O*-Bis(trimethylsilyl)trifluoroacetamide with 1% trimethylsilyl chloride (TMCS) (vol/vol) was added to each sample, vortexed for 5 min, incubated at 70°C for 1 h for the silylation process, and then rested at room temperature and in a dark environment for 30 min. At the end of the procedure, 100 µL of heptane containing 20 ppm of the internal standard pentadecanoic acid was added to each analysis vial. Two quality control samples were prepared from aliquots of each sample, and both followed the same sample preparation procedure, and they were injected every five samples, ensuring the assembly of the analytical quality control, aiming to observe the robustness and reproducibility of the chromatographic (97–99). Two blank samples prepared with the metabolic extraction solvent were injected at the beginning, middle, and end of the sample sequence. Sample analysis was conducted on a gas chromatography system coupled to a quadrupole mass spectrometer (GCMS-QP2020 NX) from Shimadzu Co. (Kyoto, Japan).

For separation, 1 µL of the sample was loaded onto a DB5-MS column (30 m × 0.25 mm, 0.25 um, Restek). The sample was injected in splitless mode at a flow of 20 mL/min of helium gas, and the carrier gas was delivered at a constant flow of 1.36 mL/min. The initial temperature of the column was initially maintained at 80°C and then gradually increased at a rate of 15°C/min until reaching the final temperature of 300°C, and maintained at this same temperature for 8 min before cooling it. The temperatures of the injector, transfer line, source filament, and the quadrupole were maintained at 280, 200, 150, and 150°C, respectively. The system operated in full scan mode (*m/z* 40–650) at a rate of 3 spectra/s and with the electron ionization (EI) configured at 70 eV. Then, a closed retention time (time-restricted feeding [TRF]) method was applied to reduce the retention time (TR) of the entire analysis (98, 99). Instrument control, data acquisition, and data processing were performed by LabSolutions software (GCMS version 4.5, Shimadzu Co., Japan), which allows real-time control of each analyte analyzed for the identification of metabolites in mode YES and Scan.

For analyses in Scan mode, the detected metabolites were processed to create a unified matrix of variables based on the different charge states, adducts, and groups of the same analytes across all samples, and for this purpose, the GCMS Solution software was used (v.3.30), NIST 17 MASS (v.1.00.1) and GCMS Smart Metabolite (v.3.01), all developed by Shimadzu Co. The software was configured in the most efficient way possible to integrate and process all detected peaks, separating the equipment noise, and false peaks detected. After identifying the molecules identified by the NIST (98), and Smart Metabolite libraries, the samples were exported to Excel software (Microsoft Office) for statistical processing. Public databases available on the internet (https://www.genome.jp/kegg/ and http://www.hmdb.ca) were used for the identification and/or confirmation of GC-MS spectra.

Univariate and multivariate statistical analyses were performed in MetaboAnalyst 5.0 (http://www.metaboanalyst.ca/) following parametric algorithms such as Student’s *t*-test and ANOVA, creating multivariate models with logarithmic transformation for normalization, scaling, and matrix normalization of data. Unsupervised models, such as principal component analysis, and supervised models, such as partial least squares regression and its orthogonal regression, were constructed to observe trends and presence of outliers, and select responsible variables by the separation shown by the models, as well as the modeling of the data in heat maps, showing groups of metabolites corresponding to the metabolic characteristics of the analyzed groups.

## Data Availability

The Illumina reads were deposited on NCBI’s GenBank database under the SRA IDs SRR25184433 to SRR25184456 and BioProject accession PRJNA991440.
